# Design and synthesis of new spirooxindole candidates and their selenium nanoparticles as potential dual Topo I/II inhibitors, DNA intercalators, and apoptotic inducers

**DOI:** 10.1080/14756366.2023.2242714

**Published:** 2023-08-18

**Authors:** Samar El-Kalyoubi, Mohamed M. Khalifa, Mahmoud T. Abo-Elfadl, Ahmed A. El-Sayed, Ahmed Elkamhawy, Kyeong Lee, Ahmed A. Al-Karmalawy

**Affiliations:** a Department of Pharmaceutical Organic Chemistry, Faculty of Pharmacy, Port Said University, Port Said, Egypt; b Pharmaceutical Medicinal Chemistry & Drug Design Department, Faculty of Pharmacy (Boys), Al-Azhar University, Cairo, Egypt; c Biochemistry Department, Biotechnology Research Institute, National Research Centre, Cairo, Egypt; d Cancer Biology and Genetics Laboratory, Centre of Excellence for Advanced Sciences, National Research Centre, Cairo, Egypt; e Photochemistry Department, Chemical Industries Research Institute, National Research Centre, Giza, Egypt; f College of Pharmacy, BK21 FOUR Team and Integrated Research Institute for Drug Development, Dongguk University—Seoul, Goyang, Republic of Korea; g Department of Pharmaceutical Organic Chemistry, Faculty of Pharmacy, Mansoura University, Mansoura, Egypt; h Pharmaceutical Chemistry Department, Faculty of Pharmacy, Ahram Canadian University, 6th of October City, Giza, Egypt

**Keywords:** Molecular hybridisation, selenium nanoparticles, dual Topo I/II inhibitors, cytotoxicity, apoptosis

## Abstract

A new wave of dual Topo I/II inhibitors was designed and synthesised via the hybridisation of spirooxindoles and pyrimidines. *In situ* selenium nanoparticles (SeNPs) for some derivatives were synthesised. The targets and the SeNP derivatives were examined for their cytotoxicity towards five cancer cell lines. The inhibitory potencies of the best members against Topo I and Topo II were also assayed besides their DNA intercalation abilities. Compound **7d NPs** exhibited the best inhibition against Topo I and Topo II enzymes with IC_50_ of 0.042 and 1.172 μM, respectively. The ability of compound **7d NPs** to arrest the cell cycle and induce apoptosis was investigated. It arrested the cell cycle in the A549 cell at the S phase and prompted apoptosis by 41.02% vs. 23.81% in the control. *In silico* studies were then performed to study the possible binding interactions between the designed members and the target proteins.

## Highlights

- A new wave of dual Topo I/II inhibitors was designed and synthesised *via* the hybridisation of spirooxindoles and pyrimidines.
- *In situ* selenium nanoparticles (SeNPs) for some derivatives were synthesised.
- Cytotoxicity, Topo I and Topo II inhibitory assays, and DNA intercalation abilities were evaluated.
- Compound **7d NPs** showed the best Topo I and Topo II inhibition.
- Cell cycle arrest, apoptosis induction, and molecular docking studies were performed.

## Graphical Abstract

**Figure:**
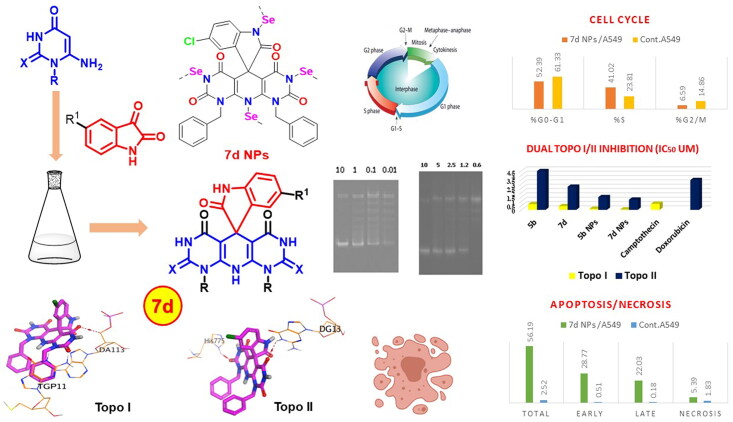

## Introduction

Multitarget therapies have, recently, attracted the medicinal chemists’ interest, especially in the scope of complicated diseases including inflammation, thrombotic disorders as well as cancer^1^^,^^2^. The progress of such diseases can be regulated by several factors^3^. The primary or acquired resistance to almost all single-target therapies is the main challenge facing the treatment of these diseases due to the activation of compensatory mechanisms^4–6^. With respect to cancer, the currently available chemotherapeutic drugs achieved remarkable success in either curing the patients or, at least, extending their lives^7^. However, targeting two or more of the diverse pathways that participate in cancer development gives a synergistic effect to the designed drug and weakens the resistance against it^4^^,^^5^. The construction of a multitarget drug is quite more difficult than the single-target one as it requires the overlapping of the pharmacophoric features needed by the multiple targets^8^^,^^9^.

Apoptosis, the process of programmed cell death, has received the researchers’ attention in the last decades and became the target for almost all anticancer agents since the progress of tumours is associated with the failure of the apoptotic security pathways in deregulated cells^10^. Therefore, apoptosis induction in cancer cells is considered an effective pathway to eradicate uncontrolled proliferating cells. Thus, targeting DNA or, even, the enzymes involved in its metabolism is nowadays among the most promising strategies for cancer treatment^11^^,^^12^.

Topoisomerases are a group of enzymes that resolve DNA topological problems by untangling the DNA duplexes^13^. The later fact potentiates the topoisomerases’ role in DNA transcription and replication^13^. These enzymes can relax the DNA supercoiling upon the formation of transient single-stranded (topoisomerase I or Topo I) or double-stranded (topoisomerase II or Topo II) splits in DNA molecules^14^. Following, the DNA segment can then pass through the formed splits releasing the DNA molecule tension. However, the high levels of Topo I and Topo II enzymes in malignant cells compared to that of normal cells make the enzymes promising objectives for cancer chemotherapy^15^. Thus, DNA Topo I and Topo II inhibitors are classified as antitumor agents that form the basis of different chemotherapy combinations and are used widely in a broad spectrum of tumours^16^.

However, targeting Topo II by Topo II inhibitors motivates a cell resistance mechanism via a decrease in the cellular Topo II levels. This leads, subsequently, to an elevation in the Topo I cellular levels with an increase in the cells’ sensitivity to Topo I inhibitors. The opposite phenomenon also occurs regarding resistance towards Topo I inhibitors^17^. Owing to this reason, targeting both Topo I and Topo II not only gives a synergistic effect but also weakens the probability of the development of resistance against such inhibitors^17^. Unfortunately, severe neutropenia and anaemia have been clinically reported after the co-application of Topo I and Topo II inhibitors^18^. An alternative approach to such a combination was the use of a dual inhibitor of both targets. Thus far, several dual Topo I/II inhibitors have been recognised^19^.

**Figure 1.**
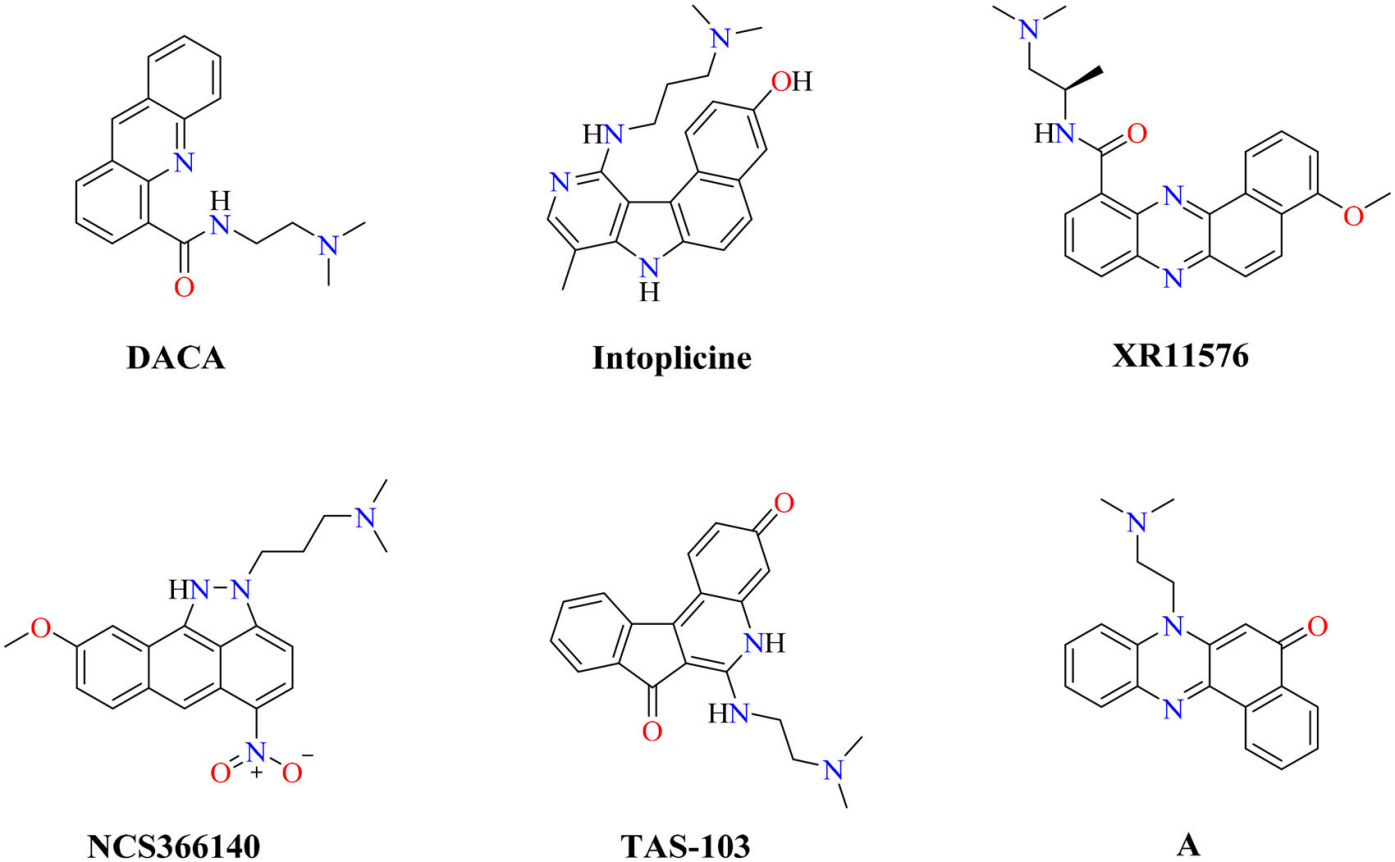
Chemical structures of some reported dual Topo I/II inhibitors.

Moreover, dual Topo I/II inhibitors are classified into either DNA intercalating or DNA non-intercalating molecules according to their mechanism of action. The DNA intercalating molecules represent the majority of well-known dual Topo I/II inhibitors. Also, DNA intercalators are molecules that possess a planer (flat) aromatic system that can be sandwiched between two adjacent DNA nucleobases causing structural modifications to the DNA molecule^11^. Structural modification for the DNA intercalating molecules by linking their flat aromatic system to an amino group enables them to interact with Topo I/II enzymes. Figure 1 illustrates some reported Topo I/II inhibitors with potent *in vitro* anticancer effects against different types of cancers^17^^,^^20–26^.

The spirooxindole-based structures are among the most vital nitrogen-containing compounds, particularly in the treatment of cancer due to the structural rigidity attributed to the spirocarbon present in these heterocycles^27–29^. However, spirooxindoles were reported to inhibit Topo I as well as Topo II enzymes^30^^,^^31^. In accordance with this, high interest was directed towards either discovering novel anticancer spirooxindole-based members or finding a simple and novel way to synthesise spirooxindoles. On the other hand, pyrimidine-based scaffolds also have a clear role in building several biologically active compounds, particularly in the field of cancer treatment^32^. The biological significance of pyrimidines may be ascribed to the ability of the nitrogen atom to bind with DNA by hydrogen bonding^33^. However, several pyrimidine-based structures were reported to inhibit topoisomerases including, benzo[4,5]thieno[2,3-*d*]pyrimidines^34^, thiazolo[3,2-*a*]pyrimidines^35^, triazolo[4,3-*a*]pyrimidines^36^, imine-pyrazolopyrimidinones^37^, pyrimido[5,4-*b*]indoles^38^, pyrazolo[3,4-*d*]pyrimidines^38^, thieno[2,3-*d*]pyrimidine^34^, and pyrido[2,3-*d*:6,5-*d*′]dipyrimidine^39^.

**Figure 2.**
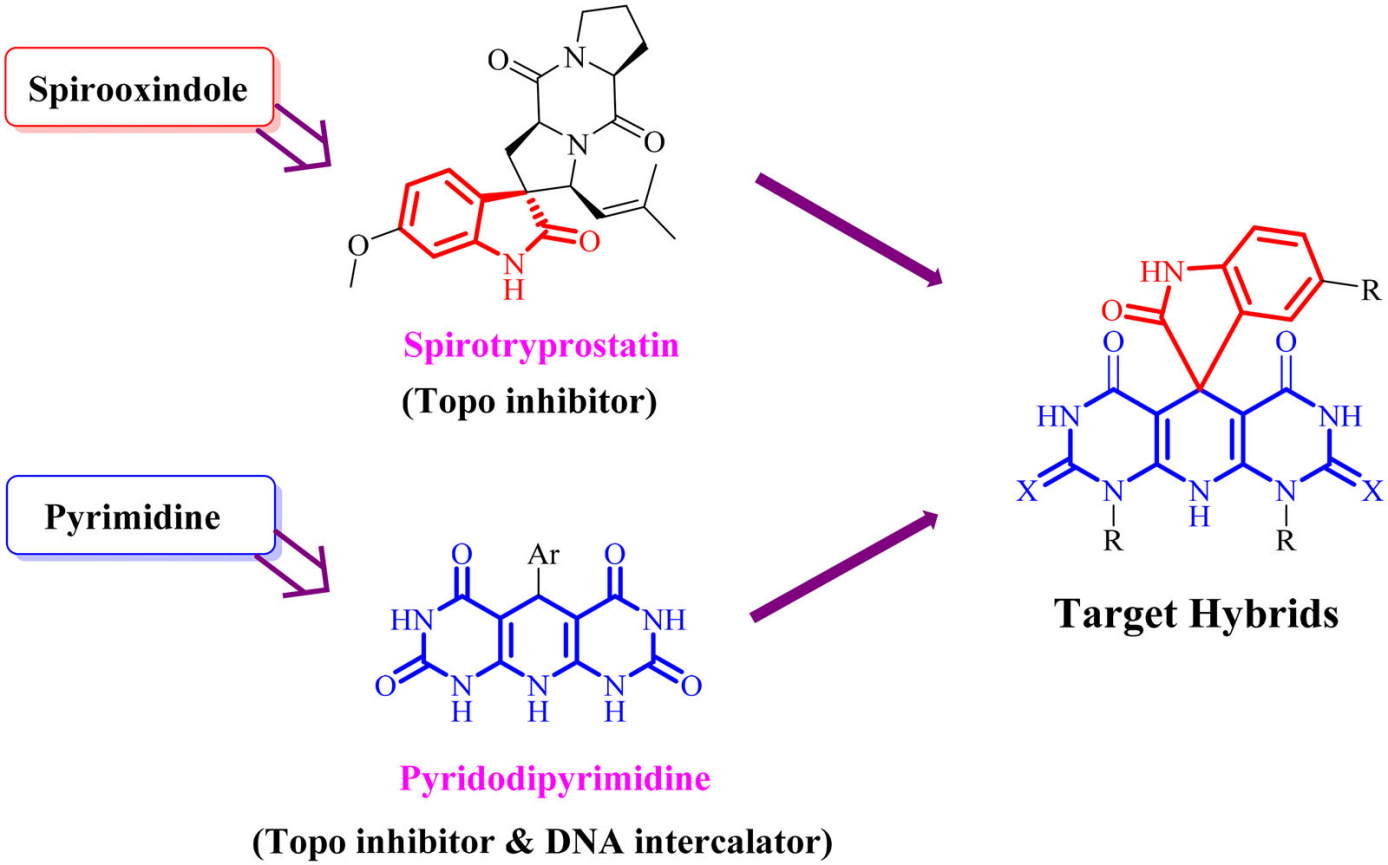
Strategy for building the target hybrids.

Based on the importance of spirooxindoles and pyrimidines, we decided to design a novel hybrid of DNA intercalating Topo I/II inhibitors based on the spiro[indoline-3,5′-pyrido[2,3-*d*:6,5-*d*′]dipyrimidine] scaffold hoping to complete our efforts in searching for potent anticancer agents (Figure 2).

Selenium (Se) is a crucial trace element required for different cellular functions through incorporation into selenoproteins^40^. The significance of selenium nanoparticles (SeNPs) stems from their interaction with proteins, bioavailability, low toxicity, and biocompatibility related to organic and inorganic selenium. At high doses, SeNPs have been shown to have potent anticancer activity. SeNPs have excellent optoelectronic and semiconducting properties and are used in various applications ranging from photovoltaic cells, semiconductors, rectifiers, and copiers to biology and medicine^41^. SeNPs have been used in biomedical applications such as drug and targeted gene delivery, anti-inflammatory, antibacterial, and anticancer^42^. Many reports described the synthesis of SeNPs using different methods such as microwave-assisted synthesis, laser ablation, chemosynthetic reduction, electrodeposition, solvothermal, and green synthesis, all as examples of green synthesis. However, acidic pH, harsh chemical conditions, and high temperatures limit their use in biomedical applications^43^.

### The molecular design rationale

**Figure 3.**
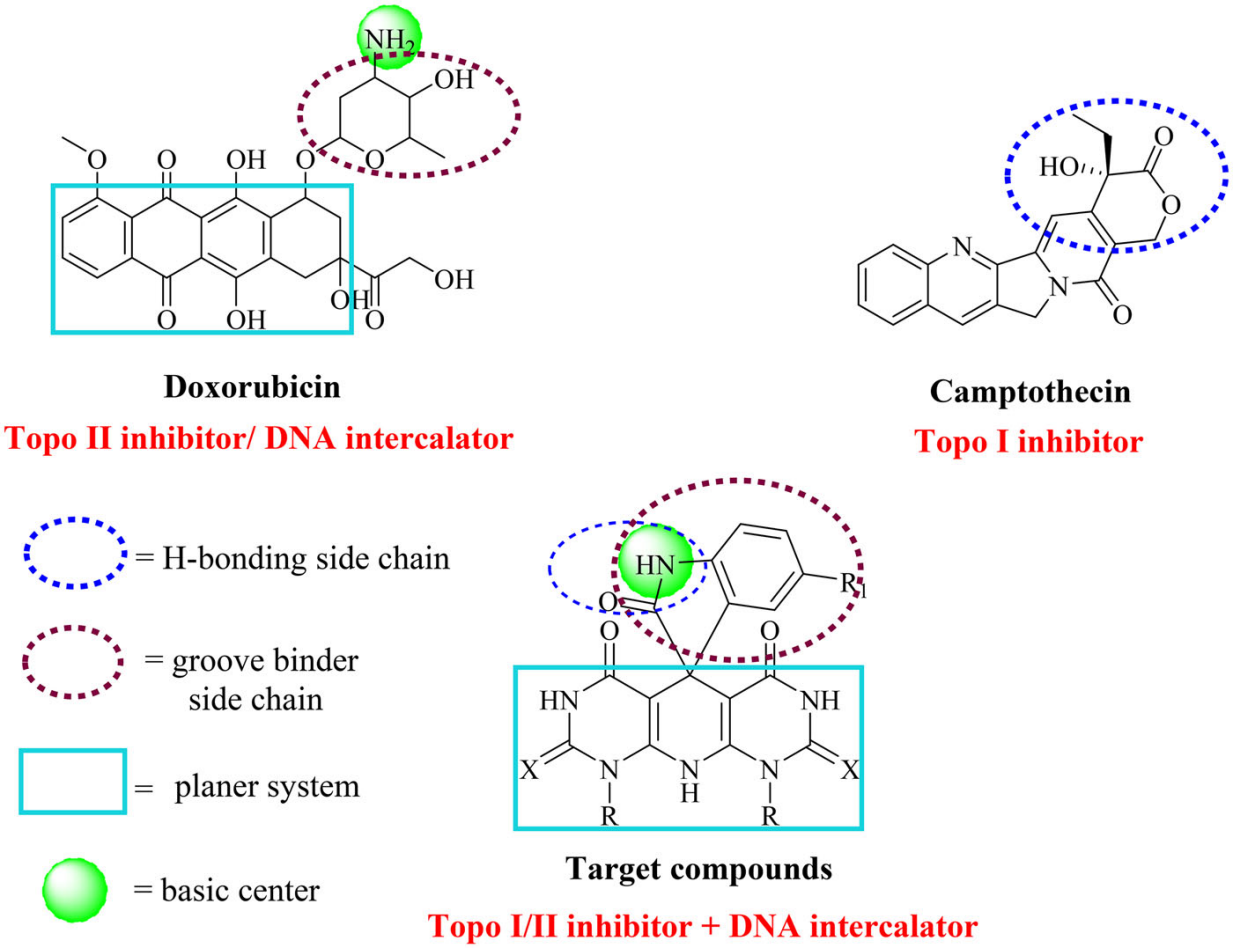
The molecular design rationale of the new DNA intercalating and Topo I/II inhibitors.

The design of a new wave of compounds that dual-target Topo I and II enzymes and intercalate onto DNA depends basically upon the overlap between the reported pharmacophoric features of both Topo inhibitors and DNA intercalators. With this respect, we designed a new series of spiro[indoline-3,5′-pyrido[2,3-*d*:6,5-*d*′]dipyrimidine] candidates that fulfilled the requirements needed for a considerable binding with both receptors. Camptothecin, a Topo I inhibitor, and doxorubicin (Dox), a potent Topo II inhibitor and DNA intercalator, were our guides in the recent study. As so, the newly designed members achieved the following three features: (A) a planer heteroaromatic pyridodipyrimidine system to intercalate in between the DNA base pairs; (B) an NH basic centre to bind the negatively charged phosphate group of the sugar moiety; (C) a groove binder side chain to fit the DNA minor groove; and (D) a hydrogen bonding side chain to interact with both enzymes (Figure 3).

## Materials and methods

### Chemistry

#### General

SMP 30 apparatus was applied to record the melting point of the obtained congeners and the results were uncorrected. The progress of the synthetic steps was checked by the pre-coated silica gel plates (Merck 60 F_254_, Darmstadt, Germany) followed by visualising the applied spots with a UV lamp (254 nm). ^1^H NMR and ^13^C NMR (400 and 100 MHz, respectively) spectra were carried out by a Bruker NMR spectrometer (Billerica, MA). DMSO-*d*_6_ was the solvent used in the NMR analysis. While mass spectra were obtained using Varian MAT 311-A (70 eV).

#### Materials

The starting compounds, 6-aminouracil derivatives (**1a**–**e**) were synthesised following the reported protocol^44–46^. Reagents and chemicals were supplied either from the USA or from Aldrich Chemicals Co. (St. Louis, MO). However, some reagents were obtained from commercial sources.

#### 1′,9′-Diethyl-1′H-spiro[indoline-3,5′-pyrido[2,3-d:6,5-d′]dipyrimidine]-2,2′,4′,6′,8′(3′H,7′H,9′H,10′H)-pentaone (3)

A mixture of 6-amino-1-ethyluracil (**1b)** (1.29 mmol) and isatin (**2**) (0.65 mmol) was heated under reflux for 2 h with the addition of a few traces of glacial acetic acid (6 mL). Next, the developed precipitate was filtered off. The collected product was then washed with methanol followed by a recrystallisation from DMF.

Yield: 90%; m.p. >300 °C; ^1^H NMR (400 MHz, DMSO) *δ* 11.84 (s, 1H, NH), 10.88 (s, 1H, NH), 10.72 (s, 1H, NH), 9.20 (s, 1H, NH), 7.30 (d, *J* = 8.1 Hz, 1H, ArH), 7.21–7.14 (m, 1H, ArH), 7.00–6.87 (m, 2H, ArH), 4.14–3.80 (m, 4H, 2CH_2_), 1.18 (t, *J* = 6.6 Hz, 6H, 2CH_3_); ^13^C NMR (100 MHz, DMSO) *δ* 181.76, 160.64, 157.68, 152.63, 150.38, 149.82, 146.22, 135.70, 128.11, 125.99, 123.74, 120.89, 116.80, 98.25, 83.40, 50.24, 38.27, 36.45, 13.76, 13.61 ppm. MS: *m/z* (%) = 422 (M^+^, 20), 410 (66), 359 (82), 282 (53), 186 (50), 185 (39), 121 (42), 106 (43), 105 (46), 99 (46), 91 (41), 83 (100), 77 (59); Anal. Calcd. for C_20_H_18_N_6_O_5_ (422.40): C, 56.87; H, 4.30; N, 19.90, found: C, 57.19; H, 4.41; N, 20.13.

#### General method for the preparation of 1′,9′-dialkyl-5-nitro-1′H-spiro[indoline-3,5′-pyrido[2,3-d:6,5-d′]dipyrimidine]pentaones and/or dithioxotrione (5a–e)

A solution of 6-aminouracils **1a**–**e** (1.5 mmol) with 5-nitroisatin (**4**) (0.75 mmol) was refluxed for 2–2.5 h, with a few drops of glacial acetic acid (7 mL). Then, the collected precipitate was rinsed with methanol and recrystallised from DMF to yield the target members **5a**–**e** in good to excellent yields.

##### 1′,9′-Dimethyl-5-nitro-1′H-spiro[indoline-3,5′-pyrido[2,3-d:6,5-d′]dipyrimidine]-2,2′,4′,6′,8′(3′H,7′H,9′H,10′H)-pentaone (5a)

Yield: 84%; m.p. >300 °C; ^1^H NMR (400 MHz, DMSO) *δ* 11.72 (s, 1H, NH), 11.13 (s, 1H, NH), 10.82 (s, 1H, NH), 9.91 (s, 1H, NH), 8.11 (dd, *J* = 9.0, 2.4 Hz, 1H, ArH), 7.74 (s, 1H, ArH), 7.51 (d, *J* = 9.0 Hz, 1H, ArH), 3.46 (s, 3H, CH_3_), 3.34 (s, 3H, CH_3_); ^13^C NMR (100 MHz, DMSO) *δ* 181.44, 160.98, 158.28, 154.36, 151.22, 150.33, 147.21, 143.42, 141.93, 124.58, 122.62, 122.04, 117.98, 98.46, 84.46, 50.45, 30.75, 29.71 ppm. MS: *m/z* (%) = 439 (M^+^, 30), 432 (58), 430 (41), 427 (54), 419 (49), 418 (55), 412 (71), 409 (86), 359 (62), 353 (48), 324 (44), 275 (61), 273 (63), 245 (74), 229 (51), 183 (61), 132 (71), 91 (65), 89 (92), 58 (81), 50 (100); Anal. Calcd. for C_18_H_13_N_7_O_7_ (439.34): C, 49.21; H, 2.98; N, 22.32, found: C, 49.40; H, 3.19; N, 22.54.

##### 1′,9′-Diethyl-5-nitro-1′H-spiro[indoline-3,5′-pyrido[2,3-d:6,5-d′]dipyrimidine]-2,2′,4′,6′,8′(3′H,7′H,9′H,10′H)-pentaone (5b)

Yield: 87%; m.p. >300 °C; ^1^H NMR (400 MHz, DMSO) *δ* 11.75 (s, 1H, NH), 11.15 (s, 1H, NH), 10.84 (s, 1H, NH), 9.83 (s, 1H, NH), 8.12 (dd, *J* = 9.1, 2.5 Hz, 1H, ArH), 7.68 (d, *J* = 2.5 Hz, 1H, ArH), 7.55 (d, *J* = 9.1 Hz, 1H, ArH), 4.17–3.78 (m, 4H, 2CH_2_), 1.19 (m, 6H, 2CH_3_). ^13^C NMR (100 MHz, DMSO) *δ* 181.05, 160.52, 157.73, 152.96, 150.29, 149.58, 145.79, 142.92, 141.67, 124.15, 122.00, 121.68, 117.52, 98.32, 84.33, 49.93, 38.44, 36.79, 13.71 ppm. MS: *m/z* (%) = 467 (M^+^, 12), 427 (43), 388 (40), 371 (42), 249 (100), 245 (45), 186 (40), 103 (73), 97 (83); Anal. Calcd. for C_20_H_17_N_7_O_7_ (467.40): C, 51.40; H, 3.67; N, 20.98, found: C, 51.67; H, 3.84; N, 21.19.

##### 1′,9′-Dimethyl-5-nitro-2′,8′-dithioxo-2′,3′,8′,9′-tetrahydro-1′H-spiro[indoline-3,5′-pyrido[2,3-d:6,5-d′]dipyrimidine]-2,4′,6′(7′H,10′H)-trione (5c)

Yield: 85%; m.p. >300 °C; ^1^H NMR (400 MHz, DMSO) *δ* 12.61 (s, 1H, NH), 12.39 (s, 1H, NH), 11.96 (s, 1H, NH), 10.12 (s, 1H, NH), 8.13 (d, *J* = 9.0 Hz, 1H, ArH), 7.85 (d, *J* = 2.3 Hz, 1H, ArH), 7.59 (d, *J* = 9.4 Hz, 1H, ArH), 3.96 (s, 3H, CH_3_), 3.73 (s, 3H, CH_3_). ^13^C NMR (100 MHz, DMSO) *δ* 180.03, 175.83, 174.54, 161.58, 161.39, 155.22, 154.50, 146.72, 143.18, 124.52, 122.51, 120.61, 118.16, 89.91, 84.69, 50.31, 36.64, 35.92 ppm. MS: *m/z* (%) = 471 (M^+^, 34), 455 (53), 447 (40), 364 (36), 307 (40), 284 (75), 270 (100), 258 (56), 233 (50), 205 (63), 156 (67), 135 (61); Anal. Calcd. for C_18_H_13_N_7_O_5_S_2_ (471.47): C, 45.86; H, 2.78; N, 20.80, found: C, 46.03; H, 3.04; N, 21.07.

##### 1′,9′-Dibenzyl-5-nitro-1′H-spiro[indoline-3,5′-pyrido[2,3-d:6,5-d′]dipyrimidine]-2,2′,4′,6′,8′(3′H,7′H,9′H,10′H)-pentaone (5d)

Yield: 81%; m.p. >300 °C; ^1^H NMR (400 MHz, DMSO) *δ* 11.93 (s, 1H, NH), 11.38 (s, 1H, NH), 11.03 (s, 1H, NH), 10.05 (s, 1H, NH), 8.11–8.06 (m, 1H, ArH), 7.72–7.68 (m, 1H, ArH), 7.42–7.25 (m, 11H, ArH), 5.45–4.92 (m, 4H, 2CH_2_). ^13^C NMR (100 MHz, DMSO) *δ* 180.98, 160.85, 157.99, 153.41, 150.48, 149.96, 146.40, 143.14, 142.76, 137.54, 136.38, 136.14, 128.81, 128.40, 127.65, 126.44, 126.33, 126.28, 124.53, 124.42, 121.99. 121.87, 121.63, 117.61, 117.32, 98.70, 84.51, 50.23, 46.12, 44.33 ppm. MS: *m/z* (%) = 591 (M^+^, 33), 583 (40), 499 (100), 498 (39), 481 (55), 428 (48), 381 (43), 342 (53), 326 (61), 310 (54), 287 (54), 263 (45), 198 (49), 166 (50), 156 (56); Anal. Calcd. for C_30_H_21_N_7_O_7_ (591.54): C, 60.91; H, 3.58; N, 16.58, found: C, 60.78; H, 3.71; N, 16.80.

##### 5-Nitro-1′H-spiro[indoline-3,5′-pyrido[2,3-d:6,5-d′]dipyrimidine]-2,2′,4′,6′,8′(3′H,7′H,9′H,10′H)-pentaone (5e)^47^

Yield: 85%; m.p. >300 °C; ^1^H NMR (400 MHz, DMSO) *δ* 11.85 (s, 1H, NH), 11.38 (s, 2H, NH), 10.84 (s, 1H, NH), 10.54 (s, 1H, NH), 10.10 (s, 1H, NH), 8.05 (dd, *J* = 9.0, 2.6 Hz, 1H), 7.59 (s, 1H, ArH), 7.22 (d, *J* = 9.0 Hz, 1H, ArH). ^13^C NMR (100 MHz, DMSO) *δ* 181.12, 162.36, 159.15, 153.24, 151.87, 150.80, 146.29, 142.88, 141.96, 124.65, 122.63, 122.46, 117.26, 97.93, 83.62, 49.42 ppm. MS: *m/z* (%) = 411 (M^+^, 45), 402 (61), 378 (43), 362 (41), 224 (29), 203 (32), 196 (33), 182 (40), 171 (46), 167 (55), 166 (62), 149 (67), 147 (100), 145 (43), 144 (75), 119 (46), 84 (61), 69 (79); Anal. Calcd. for C_16_H_9_N_7_O_7_ (411.29): C, 46.73; H, 2.21; N, 23.84, found: C, 46.97; H, 2.43; N, 23.79.

#### General method for the preparation of 1′,9′-dialkyl-5-chloro-1′H-spiro[indoline-3,5′-pyrido[2,3-d:6,5-d′]dipyrimidine]pentaones and/or dithioxotrione (7a–e)

6-Aminouracils **1a**–**e** (1.5 mmol) and 5-chloroisatin (**6**) (0.75 mmol) were heated in the presence of glacial acetic acid (7 mL) for 2–2.5 h at reflux. Filtration was used to collect the precipitate, which was then washed with methanol, recrystallised from DMF, and dried in the oven to yield the desired **7a**–**e** in excellent yields.

##### 5-Chloro-1′,9′-dimethyl-1′H-spiro[indoline-3,5′-pyrido[2,3-d:6,5-d′]dipyrimidine]-2,2′,4′, 6′,8′(3′H,7′H,9′H,10′H)-pentaone (7a)

Yield: 91%; m.p. >300 °C; ^1^H NMR (400 MHz, DMSO) *δ* 11.52 (s, 1H, NH), 10.94 (s, 1H, NH), 10.78 (s, 1H, NH), 9.38 (s, 1H, NH), 7.29 (d, *J* = 8.7 Hz, 1H, ArH), 7.24 (dd, *J* = 8.7, 2.1 Hz, 1H, ArH), 7.04 (s, 1H, ArH), 3.38 (s, 3H, CH_3_), 3.32 (s, 3H, CH_3_). ^13^C NMR (100 MHz, DMSO) *δ* 181.62, 160.97, 158.19, 154.30, 151.33, 150.42, 147.29, 135.19, 128.71, 127.69, 126.28, 123.39, 119.08, 98.00, 83.61, 50.78, 30.73, 29.49 ppm. MS: *m/z* (%) = 430 (M^+^+2, 8), 428 (M^+^, 29), 426 (35), 414 (43), 405 (49), 401 (83), 385 (27), 383 (76), 369 (100), 366 (94), 301 (55), 249 (54), 244 (98), 242 (62), 216 (61), 215 (54), 206 (69), 165 (82), 159 (16), 157 (58); Anal. Calcd. for C_18_H_13_ClN_6_O_5_ (428.79): C, 50.42; H, 3.06; N, 19.60, found: C, 50.71; H, 3.28; N, 19.79.

##### 5-Chloro-1′,9′-diethyl-1′H-spiro[indoline-3,5′-pyrido[2,3-d:6,5-d′]dipyrimidine]-2,2′,4′, 6′,8′(3′H,7′H,9′H,10′H)-pentaone (7b)

Yield: 92%; m.p. >300 °C; ^1^H NMR (400 MHz, DMSO) *δ* 11.53 (s, 1H, NH), 10.94 (s, 1H, NH), 10.77 (s, 1H, NH), 9.32 (s, 1H, NH), 7.34 (d, *J* = 8.8 Hz, 1H, ArH), 7.27 (dd, *J* = 8.8, 2.3 Hz, 1H, ArH), 6.93 (s, 1H, ArH), 3.95 (m, 4H, 2CH_2_), 1.18 (t, *J* = 6.7 Hz, 6H, 2CH_3_). ^13^C NMR (100 MHz, DMSO) *δ* 181.78, 160.97, 158.13, 153.52, 150.83, 150.14, 146.49, 135.30, 128.70, 127.60, 125.98, 123.27, 119.05, 98.16, 83.74, 50.60, 38.82, 36.99, 14.20, 14.04 ppm. MS: *m/z* (%) = 458 (M^+^+2, 14), 456 (M^+^, 31), 368 (54), 362 (44), 359 (43), 358 (42), 353 (24), 351 (64), 342 (28), 340 (76), 339 (77), 313 (50), 233 (52), 219 (61), 218 (63), 199 (56), 197 (92), 196 (69), 188 (46), 184 (15), 183 (52), 182 (92), 163 (100), 160 (97), 128 (45), 92 (77), 72 (52); Anal. Calcd. for C_20_H_17_ClN_6_O_5_ (456.84): C, 52.58; H, 3.75; N, 18.40, found: C, 52.75; H, 3.89; N, 18.63.

##### 5-Chloro-1′,9′-dimethyl-2′,8′-dithioxo-2′,3′,8′,9′-tetrahydro-1′H-spiro[indoline-3,5′-pyrido [2,3-d:6,5-d′]dipyrimidine]-2,4′,6′(7′H,10′H)-trione (7c)

Yield: 89%; m.p. >300 °C; ^1^H NMR (400 MHz, DMSO) *δ* 12.27 (s, 1H, NH), 11.95 (s, 1H, NH), 11.80 (s, 1H, NH), 9.61 (s, 1H, NH), 7.61 (s, 1H, ArH), 7.38–6.99 (m, 2H, ArH), 3.72 (s, 3H, CH_3_), 3.67 (s, 3H, CH_3_). ^13^C NMR (100 MHz, DMSO) *δ* 180.16, 174.51, 172.00, 161.55, 161.36, 158.90, 155.14, 154.47, 142.36, 134.39, 133.50, 128.64, 127.86, 89.87, 84.19, 50.49, 36.60, 35.88 ppm. MS: *m/z* (%) = 462 (M^+^+2, 13), 460 (M^+^, 27), 405 (11), 403 (38), 354 (37), 228 (49), 221 (32), 219 (80), 193 (61), 192 (18), 190 (66), 189 (30), 187 (80), 185 (25), 183 (100), 182 (62), 178 (52), 164 (79), 159 (70), 153 (68), 146 (60), 138 (55), 123 (57), 86 (77); Anal. Calcd. for C_18_H_13_ClN_6_O_3_S_2_ (460.91): C, 46.91; H, 2.84; N, 18.23, found: C, 47.18; H, 3.01; N, 18.45.

##### 1′,9′-Dibenzyl-5-chloro-1′H-spiro[indoline-3,5′-pyrido[2,3-d:6,5-d′]dipyrimidine]-2,2′,4′, 6′,8′(3′H,7′H,9′H,10′H)-pentaone (7d)

Yield: 88%; m.p. >300 °C; ^1^H NMR (400 MHz, DMSO) *δ* 11.71 (s, 1H, NH), 11.18 (s, 1H, NH), 10.94 (s, 1H, NH), 9.37 (s, 1H, NH), 7.39–7.19 (m, 12H, ArH), 6.89 (dd, 1H, ArH), 5.09 (s, 2H, CH_2_), 4.89 (s, 2H, CH_2_). ^13^C NMR (100 MHz, DMSO) *δ* 181.00, 160.89, 157.80, 153.68, 150.49, 149.96, 146.57, 144.78, 137.66, 136.44, 136.17, 134.65, 134.34, 128.63, 128.23, 127.62, 127.47, 127.31, 126.81, 126.38, 126.23, 122.71, 118.56, 118.32, 97.98, 81.94, 50.32, 46.05, 42.54 ppm. MS: *m/z* (%) = 582 (M^+^+2, 22), 580 (M^+^, 36), 575 (56), 565 (62), 560 (50), 555 (54), 548 (51), 543 (100), 484 (61), 466 (78), 453 (56), 348 (54), 324 (58), 326 (45), 321 (87), 305 (85), 304 (74), 295 (54), 267 (53), 258 (48), 256 (72), 217 (63); Anal. Calcd. for C_30_H_21_ClN_6_O_5_ (580.99): C, 62.02; H, 3.64; N, 14.47, found: C, 61.97; H, 3.80; N, 14.71.

##### 5-Chloro-1′H-spiro[indoline-3,5′-pyrido[2,3-d:6,5-d′]dipyrimidine]-2,2′,4′,6′,8′(3′H,7′H,-9′H,10′H)-pentaone (7e)^47^^,^^48^

Yield: 93%; m.p. >300 °C; ^1^H NMR (400 MHz, DMSO) *δ* 11.88 (s, 1H, NH), 11.06 (s, 2H, NH), 10.65 (s, 1H, NH), 10.47 (s, 1H, NH), 9.37 (s, 1H, NH), 7.22 (dd, *J* = 8.7, 2.4 Hz, 1H, ArH), 7.04 (d, *J* = 8.7 Hz, 1H, ArH), 6.80 (d, *J* = 2.4 Hz, 1H, ArH). ^13^C NMR (100 MHz, DMSO): *δ* 180.94, 161.98, 158.74, 152.86, 151.54, 150.17, 146.03, 134.55, 128.28, 126.64, 125.71, 123.18, 118.06, 96.84, 82.16, 49.18 ppm. MS: *m/z* (%) = 402 (M^+^+2, 10), 400 (M^+^, 52), 399 (23), 397 (43), 396 (7), 394 (43), 382 (7), 380 (75), 367 (42), 347 (42), 341 (25), 339 (100), 323 (68), 295 (74), 263 (57); Anal. Calcd. for C_16_H_9_ClN_6_O_5_ (400.74): C, 47.96; H, 2.26; N, 20.97, found: C, 48.12; H, 2.37; N, 21.18.

#### General method for the preparation of 1′,9′-dialkyl-5-fluoro-1′H-spiro[indoline-3,5′-pyrido[2,3-d:6,5-d′]dipyrimidine]pentaones (9a,b)

6-Aminouracils **1a,e** (1.5 mmol) and 5-fluoroisatin (**8**) (0.75 mmol) were heated in the presence of glacial acetic acid (7 mL) for 2 h under reflux. Filtration was used to collect the precipitate, which was then washed with methanol, recrystallised from DMF, and dried in the oven.

##### 5-Fluoro-1′H-spiro[indoline-3,5′-pyrido[2,3-d:6,5-d′]dipyrimidine]-2,2′,4′,6′,8′(3′H,7′H,9′H,10′H)-pentaone (9a)^47^

Yield: 83%; m.p. >300 °C; ^1^H NMR (400 MHz, DMSO) *δ* 11.81 (s, 1H, NH), 11.01 (s, 1H, NH), 10.60 (s, 2H, NH), 10.45 (s, 1H, NH), 9.17 (s, 1H, NH), 7.04 (d, 2H, ArH), 6.65 (d, 1H, ArH). ^13^C NMR (100 MHz, DMSO) *δ* 181.32, 162.43, 159.76, 159.26, 157.45, 152.95, 150.44, 146.47, 132.43, 123.20, 118.32, 115.70, 112.99, 97.22, 81.89, 49.84 ppm. MS: *m/z* (%) = 384 (M^+^, 21), 340 (30), 337 (33), 297 (40), 294 (41), 285 (55), 275 (35), 264 (37), 247 (49), 221 (40), 169 (43), 139 (100), 138 (45); Anal. Calcd. for C_16_H_9_FN_6_O_5_ (384.28): C, 50.01; H, 2.36; N, 21.87, found: C, 50.28; H, 2.54; N, 21.98.

##### 5-Fluoro-1′,9′-dimethyl-1′H-spiro[indoline-3,5′-pyrido[2,3-d:6,5-d′]dipyrimidine]-2,2′,4′,6′,8′(3′H,7′H,9′H,10′H)-pentaone (9b)

Yield: 80%; m.p. >300 °C; ^1^H NMR (400 MHz, DMSO) *δ* 11.50 (s, 1H, NH), 10.91 (s, 1H, NH), 10.76 (s, 1H, NH), 9.30 (s, 1H, NH), 7.33–7.25 (m, 1H, ArH), 7.08–7.03 (m, 1H, ArH), 6.90 (dd, *J* = 9.5, 2.7 Hz, 1H, ArH), 3.36 (s, 3H, CH_3_), 3.32 (s, 3H, CH_3_). ^13^C NMR (100 MHz, DMSO) *δ* 181.58, 161.08, 158.26, 157.78, 154.34, 151.32, 150.44, 147.42, 132.71, 123.02, 118.94, 115.76, 113.14, 97.93, 82.90, 51.07, 30.71, 29.41 ppm. MS: *m/z* (%) = 412 (M^+^, 7), 410 (29), 383 (20), 372 (45), 348 (24), 346 (45), 340 (48), 338 (45), 327 (50), 263 (34), 174 (49), 142 (100), 84 (46), 66 (44); Anal. Calcd. for C_18_H_13_FN_6_O_5_ (412.34): C, 52.43; H, 3.18; N, 20.38, found: C, 52.67; H, 3.40; N, 20.54.

### *In situ* synthesis of SeNPs using synthetic 5b and 7d heterocyclic compounds

In a conical flask, a solution of selenious acid (H_2_SeO_3_, 0.013 g, 0.01 mmol) in deionised water (80 mL) was prepared. The later solution was then heated and added to a DMSO (10 mL) solution of members **5b** or **7d** (0.01 mmol). The solutions were stirred continuously for 1 h at 60 °C. Ascorbic acid (200 µL, 40 mM) was introduced to the solutions as a catalyst. The ruby red SeNPs were then suspended. Different methods were used to confirm and characterise the formation of SeNPs as described in the following subsections.

#### Ultraviolet–visible (UV–vis) spectra

The 1st method to confirm the SeNPs formation is to characterise their UV–vis spectra. The Shimadzu spectrophotometer is used for the current experiment. These spectra were taken in the range of 400–700 nm.

#### Transmission electron microscopy (TEM)

Another confirmation method is to determine the SeNPs’ size as well as shape. High-resolution transmission electron microscopy (HRTEM), JEOL (JEM-2100 TEM; Tokyo, Japan) was applied in the current work.

#### Particle size distribution

A particle size analyser was used to determine the average diameter and size distribution (Nano-ZS, Malvern Instruments Ltd., Worcestershire, UK). Where the sample was sonicated before the assessment to measure size distribution for 30–60 min.

### Biological testing

#### Cytotoxicity of different samples on five cancer cell lines

The cytotoxic activities of the constructed members were assessed via the reported MTT method^49–52^ (Supporting information).

#### The mode of cell death of the promising cytotoxic samples (5b NPs and 7d NPs)

Herein, the acridine orange/ethidium bromide (AO/EtBr) fluorescing stain and the different modes of cell death were investigated under the fluorescent microscope using the reported methodology^53^.

#### Dual topoisomerase I/II inhibitory assay

The tested members were subjected to Topo I inhibitory activity evaluation using the previously reported method^54^. While the Topo II inhibitory assay was done according to the method described by Patra et al.^55^ (Supporting information).

#### DNA intercalation assay

The reported procedure by Atwal et al. was our guide to test the DNA intercalating ability of our candidates^56^ (Supporting information).

#### Cell cycle analysis

The propidium iodide (PI) staining strategy explained in the Supporting information was adopted in the cell cycle analysis^57^^,^^58^.

#### Cell apoptosis analysis

The applied procedure^59^ used for monitoring the apoptotic effect was mentioned in detail in the Supporting information.

### Molecular docking studies

Two molecular docking studies of the novel candidates (**3**, **5a**–**e**, **7a**–**e**, and **9a,b**) were performed using the Schrodinger suite 2021^60^ to investigate their potential activities against the human DNA in complex with either Topo I or Topo II targets, respectively. Collectively, each chemical structure was built in ChemDraw and transferred to the working window, and corrected. The structures were then energy minimised as discussed previously^61^. Each prepared series was inserted in one database together with the native ligand in each receptor. The human DNA in complex with Topo I (1T8I) and Topo II (3QX3) target receptors were extracted. Each target protein was prepared for docking as reported previously^62^. Then, two docking processes were accomplished using the corresponding database and following the default setting options and methodology as before^9^. Also, PyMOL software^63^ was applied for the visualisation of the selected poses. Moreover, two validation processes were performed by redocking each native ligand and the obtained RMSD values were <2 Å indicating a valid performance for the two docking processes^64^.

## Results and discussion

### Chemistry

**Scheme 1.**
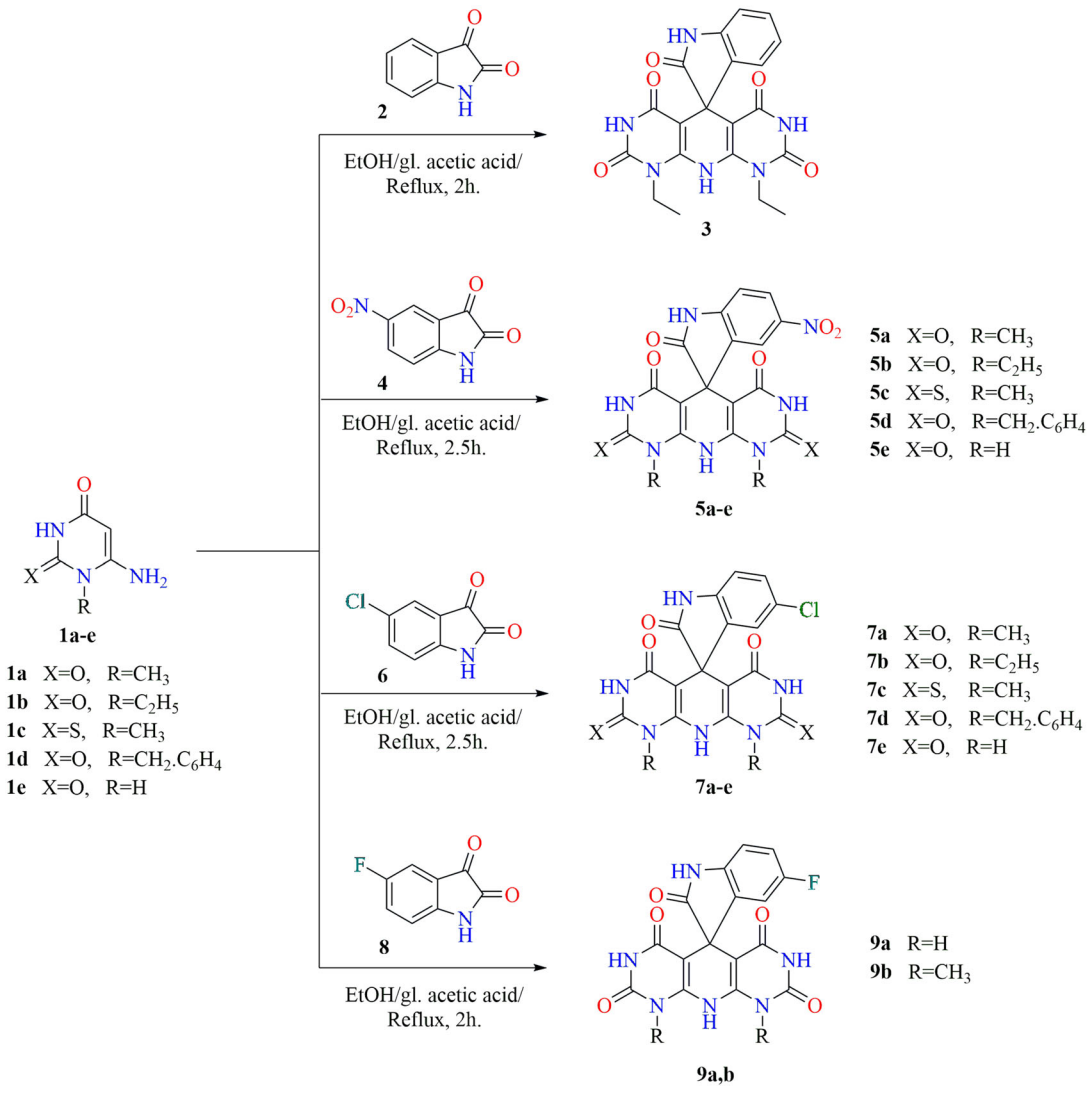
Synthesis of spirooxindole compounds **3**, **5a**–**e**, **7a**–**e**, and **9a,b**.

The spirooxindoles were synthesised by refluxing isatin with two equivalents of 6-aminouracil derivatives^44^^,^^46^^,^^65^^,^^66^ as a follow-up to our work on the uracil moiety. Bazgir and coworkers^67^ refluxed 6-aminouracils with different isatin derivatives in ethanol and *p*-TSA to get spiro[pyrimido[4,5-*b*]quinoline-5,5′-pyrrolo[2,3-*d*]pyrimidine]pentaones. While, Dong et al.^47^ synthesised spiro[indoline-3,5′-pyrido[2,3-*d*:6,5-*d*′]dipyrimidine]-pentaones (**5e**, **7e**, and **9a**) via refluxing of three-component of isatins, barbituric acid, and 6-aminouracil in the presence of (CoFe_2_O4/BC/PDA-Ni) as a catalyst for 4 h. Furthermore, Kavyani and Baharfar^48^ prepared **7e** by the reaction of 5-chloroisatin, barbituric acid, and 6-aminouracil in the presence of Fe_3_O_4_/GO/Au-Ag as a catalyst. In our project, cyclic enaminone **1a**–**e** underwent cyclocondensation with isatins to afford polyheterocyclic spiro[indoline-3,5′-pyrido[2,3-*d*:6,5-*d*′]-dipyrimidine]-pentaones and/or dithioxo-spiro[indoline-3,5′-pyrido[2,3-*d*:6,5-*d*′]dipyrimidine]-triones **3**, **5a**–**e**, **7a**–**e**, and **9a,b** by refluxing in acidic medium (Scheme 1). The investigation was extended to the uses of initiated isatin by electron acceptor group to yield more amounts of spiro derivatives and reduce the reaction time. All the resulting candidates were confirmed by spectral data ^1^H, ^13^C NMR, mass spectra, and elemental analysis. The ^1^H NMR of compounds **3**, **5a**, **5b**, **5d**, **5e**, **7a**, **7b**, **7d**, **7e**, and **9a,b** showed singlet signals characteristic for NH-3′, NH-7′ protons at *δ* 11.93–11.06 ppm, furthermore, and singlet signals at the range *δ* 11.38–10.47 ppm characteristic for NH-1 and *δ* 10.10–9.17 ppm for NH-10′. Moreover, ^1^H NMR of the thioxo-derivatives **5c** and **7c** showed characteristic singlet signals protons for NH-3′, NH-7′, NH-1, and NH-10′ at *δ* 12.61–12.27 ppm, *δ* 12.39, 11.95 ppm and at *δ* 10.12, 9.61 ppm, respectively. However, the disappearance of broad singlet signals of NH_2_-6 of uracils **1a**–**e** around *δ* 6.0 ppm proved the cyclisation and the evolution of NH_3_. ^13^C NMR spectra showed C═O signals at the range *δ* 181.98–180.03 ppm and the appearance of signals at *δ* 50.94–49.42 ppm characteristic for spiro-carbons (C-5′, 3).

**Scheme 2.**
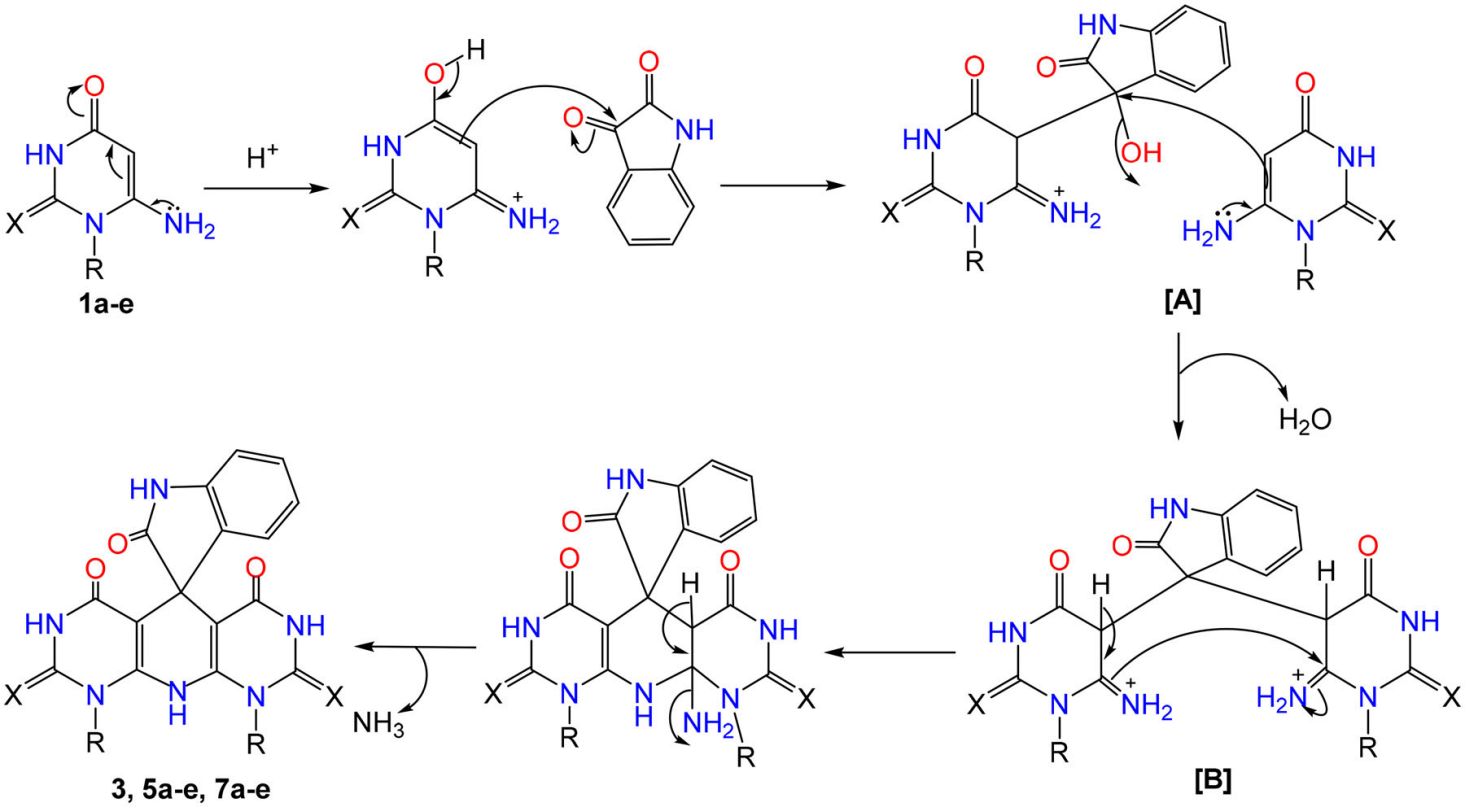
Mechanistic pathway for the synthesis of spirooxindole candidates.

The plausible mechanistic pathway for the formation of spiroindoles may be preceded as reported that the 3-position of the isatin is more reactive towards nucleophiles^68–70^. Based on this fact, intermediate [A] was formed via nucleophilic attack of the 5-position of uracil to the more reactive centre at the 3-position of isatin. The nonisolable acyclic intermediate [B] was formed via the nucleophilic attack of the 5-position of the second uracil molecule to the intermediate [A] with a loss of H_2_O. The cyclisation affording spiroindoles presumably occurred via the addition of the amino group to the C-6 of the uracil accompanied by loss of NH_3_ moiety affording the desired final products (Scheme 2).

#### In situ synthesis of SeNPs using synthetic heterocyclic compounds 5b and 7d

**Figure 4.**
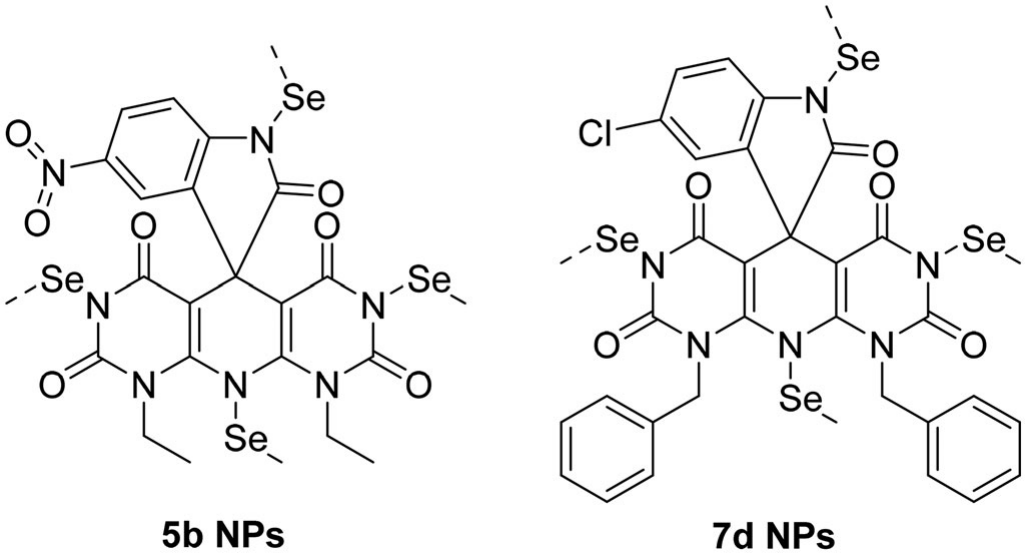
Chemical structures of heterocyclic-SeNPs (**5b NPs** and **7d NPs**).

The team’s work aimed to create new SeNPs using the synthesised compounds **5b** and **7d** having appropriate low reducing but high stabilising properties during SeNPs preparation. Accordingly, the organic heterocycle derivatives have reduced the Se^+^ cation to Se0 using an ascorbic acid catalyst, which acts as an aldehyde to form SeNPs and stabilise the nano-structure of SeNPs^71^. The organic compounds with reductive groups, such as –OH, –NH_2_, –NH, etc., can reduce Se^+^ cation. Figure 4 depicts the structure of the synthesised candidates.

**Figure 5.**
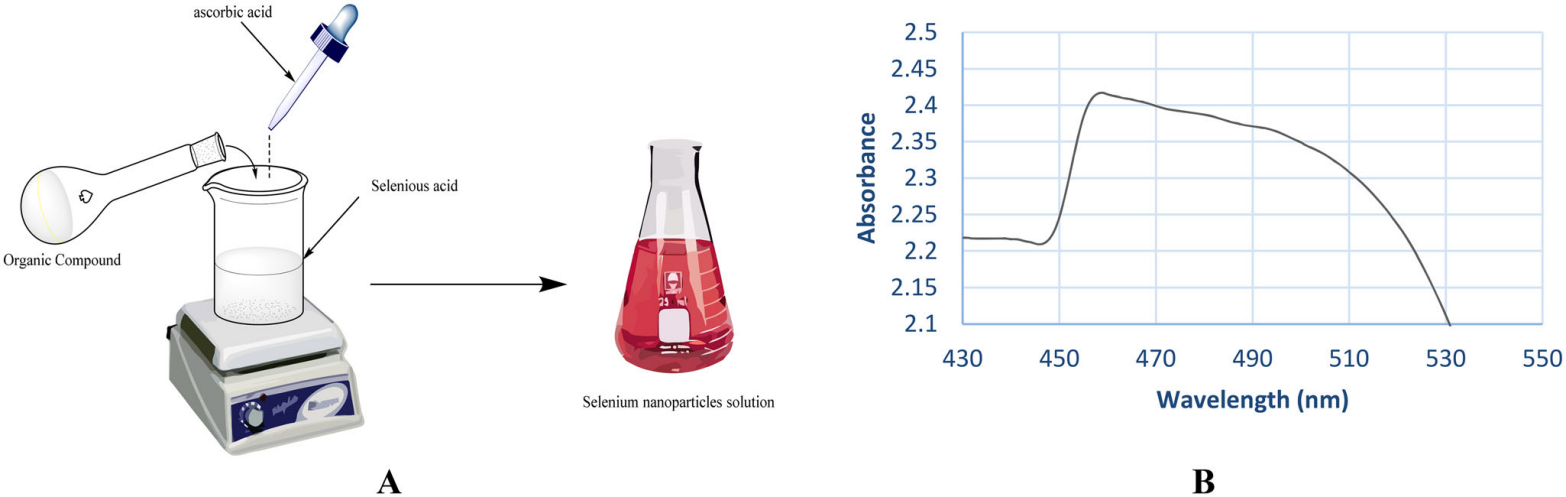
(A) Formation of NPs and (B) the UV–vis absorption spectrum of SeNPs.

The first indication for the formation of nanoparticles is the colour of the solution that turned from pale yellow to red after adding the dissolved compounds (**5b** and **7d**) in DMSO to the solution of H_2_SeO_3_ and stirring for one hour at 60 °C (Figure 5(A)). To easily monitor the formation of SeNPs, UV–vis spectroscopy was used (Figure 5(B)). The absorption peak of the selenium colloidal solution is at 460 nm, according to surface plasmon resonance peaks in absorption spectra.

##### Transmission electron microscopy

**Figure 6.**
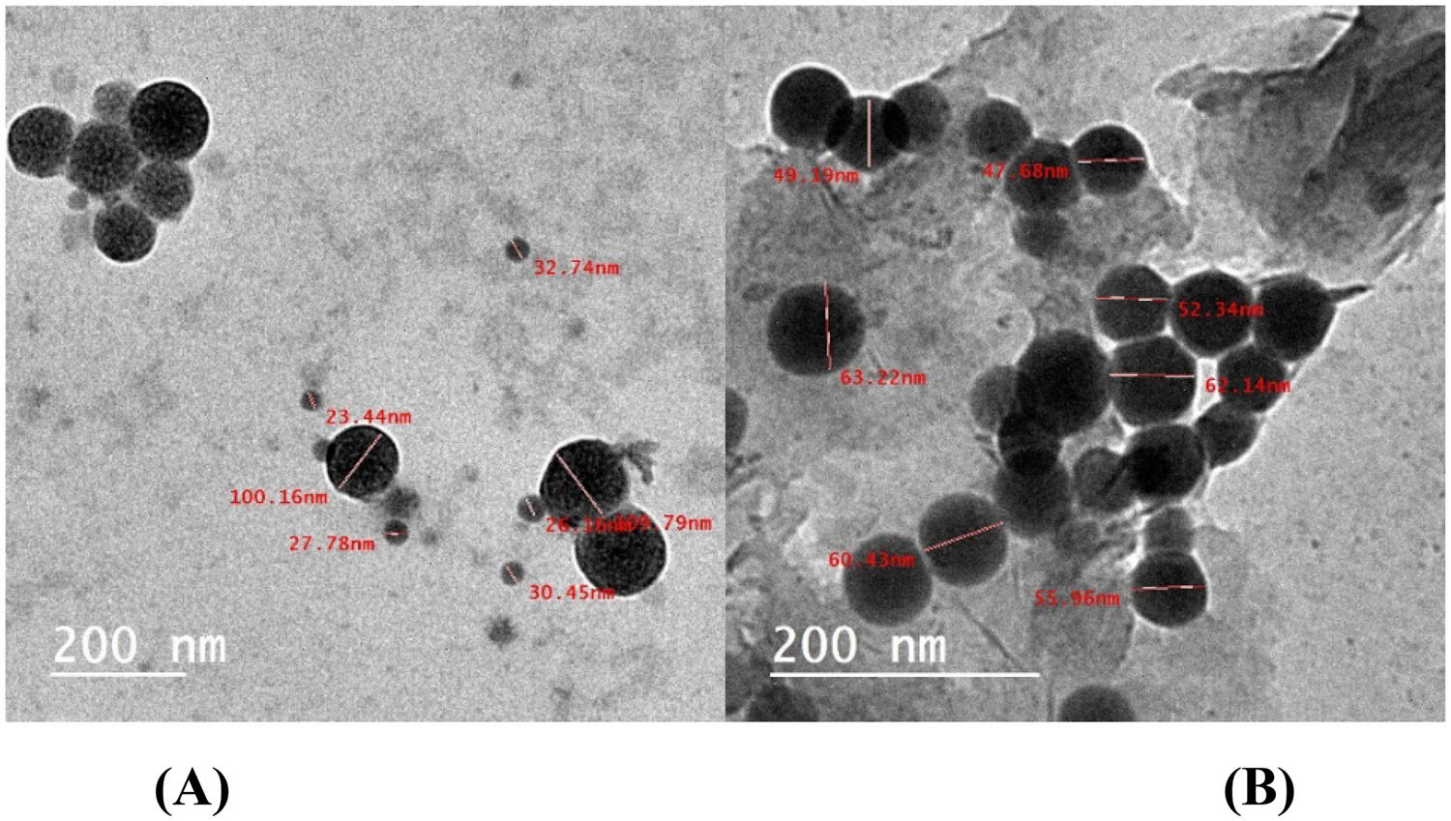
(A) TEM of SeNPs and (B) TEM of SeNPs loaded onto (**5b** and **7d**).

It is indicating that NPs were formed and dispersed without the formation of aggregations in an aqueous solution^72^. Figure 6 shows how TEM confirmed the characterisation of selenium colloidal NPs. TEM was used to examine the prepared SeNPs, either alone or loaded onto the synthesised heterocyclic compounds. Figure 6 shows the presence of formed SeNPs in the tested specimens. SeNPs in spherical shapes are depicted in Figure 6(A). Minor aggregates can be found. The size of these nanoparticles ranges from 24.98 to 60.78 nm as shown in Figure 6. When SeNPs are loaded onto the investigated compounds **1**, **2**, **3**, **4**, **5**, and **6**, they spread uniformly through both samples (Figure 6(A,B)). The synthesised compounds helped to the prevention of such agglomerates in SeNPs. It appears that the nitrogen-based compounds’ stabilising effect^73^ contributed to the existence of SeNPs separated from each other with a convenient distribution.

##### Dynamic light scattering (DLS)

**Figure 7.**
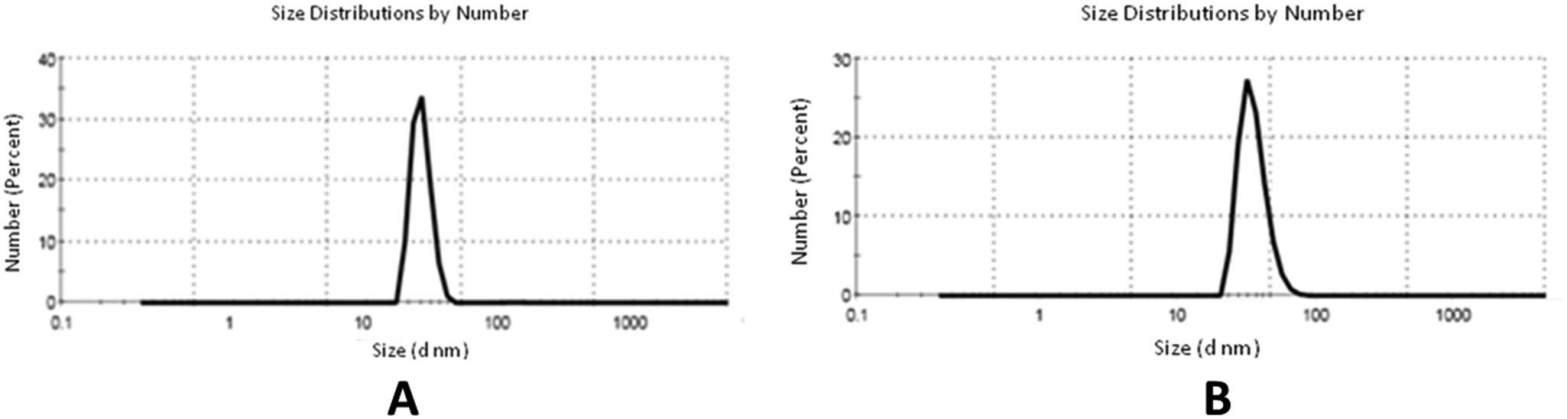
(A) Particle size from DLS of SeNPs and (B) particle size of SeNPs loaded onto (**5b** and **7d**).

The SeNPs’ particle size was estimated by DLS measurements as demonstrated in Figure 7. Results revealed the compounds’ particle size fell in the range of 30.43–65.90 nm (Table 1).

**Table 1.** The mean particle size of SeNPs.

Samples number	MPS (nm)
**5b**	30.43
**7d**	65.90

### Biological testing

#### Cytotoxicity of different samples on five cancer cell lines

The cytotoxic screening was performed using the MTT assay^74^. The assayed samples showed diverse effects on each cell line. The most sensitive tested cell lines were HepG2 and Caco-2 cells as nine out of the tested samples exhibited considerable cytotoxicity for them. However, only five tested compounds showed cytotoxic effects on the A549 cell line. Besides, the HCT-116 one was sensitive to only two samples, while the MCF7 was sensitive to one sample only. Two standard cytotoxic drugs: Dox and 5-fluorouracil (5-FU) were also applied on the same used cancer cell lines as references. The dose–response curves for each cell line are shown in Figure 4, and the respective IC_50_s are shown in Table 2.

**Table 2.** The IC_50_ of **3**, **5a-e**, **7a**–**e**, and **9a,b** compounds on five cancer cell lines and normal bone marrow cells (µg/mL).

Samples	HepG2	HCT-116	Caco-2	A-549	MCF7	Bone marrow
**3**	NA	NA	259.4 ± 9.4	291 ± 16.4	NA	–
**5a**	562.8 ± 25.5	NA	207 ± 19.4	NA	NA	–
**5b**	113.5 ± 10.1	NA	35.2 ± 4.1	NA	NA	NA
**5c**	250.3 ± 19.3	185.9 ± 19.4	247.4 ± 12.5	525.4 ± 50.6	NA	–
**5d**	421.6 ± 22.8	NA	108.5 ± 10	279.3 ± 18.9	NA	–
**5e**	146.9 ± 10.5	NA	232.8 ± 19.2	NA	NA	–
**7a**	120.3 ± 9.2	NA	112.8 ± 10.4	50.1 ± 5.9	NA	NA
**7b**	NA	NA	NA	NA	170.8 ± 22.8	–
**7c**	NA	NA	NA	NA	NA	–
**7d**	91.4 ± 11.3	NA	NA	328.6 ± 12.5	NA	NA
**7e**	89.7 ± 6.5	434.8 ± 39.5	471.9 ± 23.6	NA	NA	NA
**9a**	NA	NA	NA	NA	NA	NA
**9b**	86.4 ± 7.4	NA	492.5 ± 40.4	292.9 ± 15.4	NA	–
**Dox**	0.3 ± 0.01	1.1 ± 0.1	3.7 ± 0.2	0.04 ± 0.003	5.9 ± 0.5	0.3 ± 0.01
**5-FU**	103 ± 9.8	76.3 ± 6.8	4.4 ± 0.3	1.1 ± 0.1	39.3 ± 2.9	103 ± 9.8

NA: not available.

The data are represented as mean ± SD.

After the cytotoxicity evaluation, some compounds were selected for the synthesis of *in-situ* SeNPs. The synthesised SeNPs were confirmed using the UV–vis (spectrophotometer), TEM, and particle size distribution. On the nanoscale, the toxicity was much enhanced for samples **5b NPs** and **7d NPs**. All the IC_50_ values were around 10 μg/mL and less, except for **7d NPs** on the Caco-2 cell line, which reached 18.9 μg/mL.

The dose–response curves for the nanoform on each cell line are shown in the Supporting information, and the respective IC_50_s are shown in Table 3. The most promising cytotoxic samples were further investigated for their cytotoxic effects on normal mice bone marrow cells. The overall trending results were increasing the proliferation of the bone marrow cells at high concentrations of the samples, especially the **5b NPs** nanoform sample, which reached up to 200% compared to the untreated cells. The dose–response curves on the bone marrow cells are represented in the Supporting information.

**Table 3.** The IC_50_ of samples **5b NPs** and **7d NPs** on five cancer cell lines (µg/mL).

Samples	HepG2	HCT-116	Caco-2	A-549	MCF7
**5b NPs**	10.4 ± 1.1	10.35 ± 1.1	10.25 ± 1.01	5.8 ± 0.6	2.03 ± 0.1
**7d NPs**	3.76 ± 0.4	5.9 ± 3.4	18.9 ± 2.4	1.9 ± 0.1	NA

NA: not available.

The data are represented as mean ± SD.

#### The mode of cell death of the promising cytotoxic samples (5b NPs and 7d NPs)

**Figure 8.**
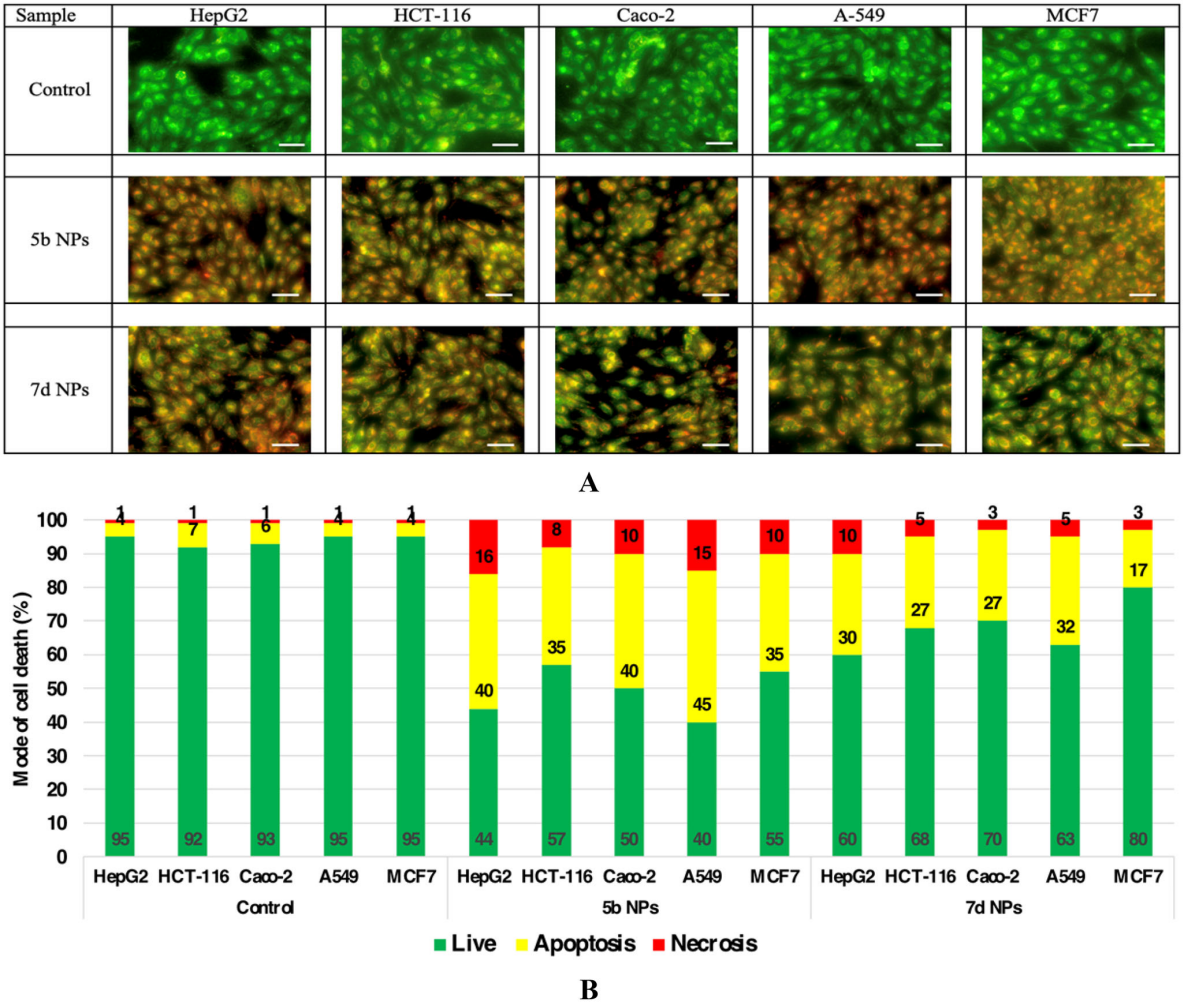
The mode of cell death of samples **5b NPs** and **7d NPs** on different cell lines. (A) The fluorescence stain using acridine orange/ethidium bromide stain. The photos show high early and late apoptotic changes with considerable necrotic changes. The scale bar is 50 µm and the magnification is ×20. (B) The % distribution of modes of cell death among different cell lines compared to their respective control cells.

Using the AO/EtBr fluorescing stain, the different modes of cell death were investigated under the fluorescent microscope. The photos of samples **5b NPs** and **7d NPs** showed high early and late apoptotic changes with considerable necrotic changes in almost all cell lines. The mode of cell death photos and the % distribution of modes among different cell lines are illustrated in Figure 8.

**Figure 9.**
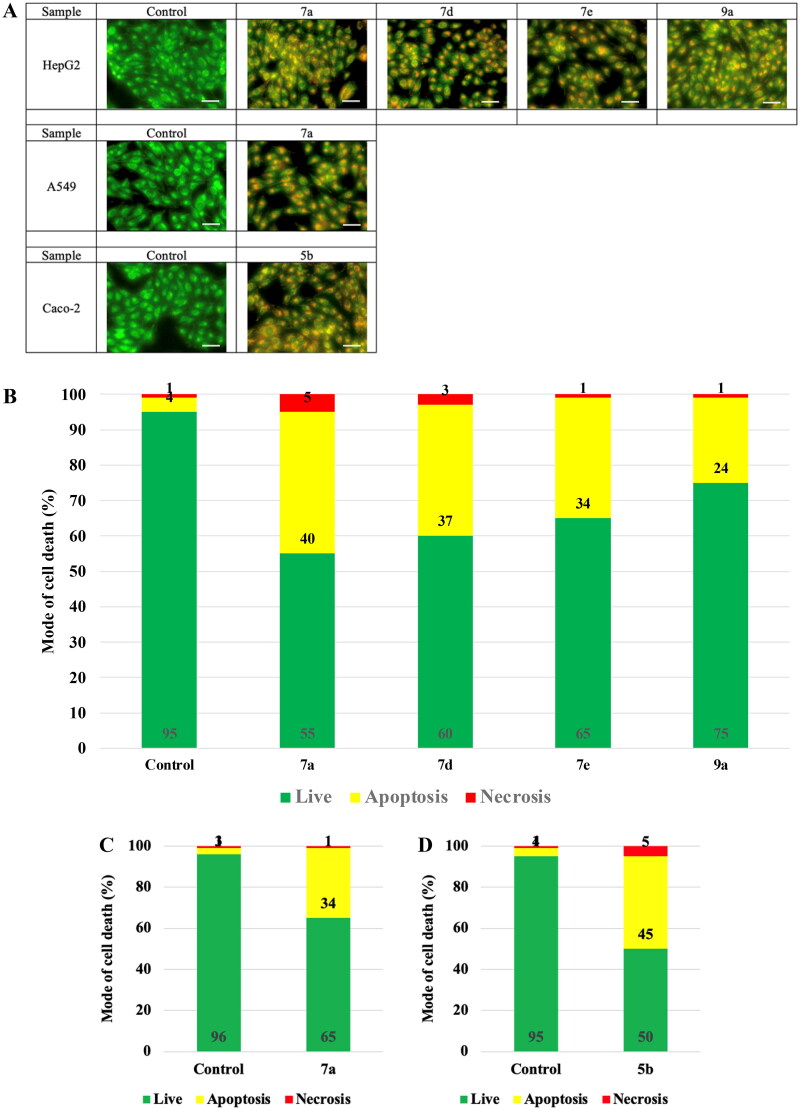
The mode of cell death of the most promising samples on different cell lines. (A) The fluorescing stain is acridine orange/ethidium bromide. The photos show high early and late apoptotic changes with considerable necrotic changes. The scale bar is 50 µm and the magnification is ×20. The % distribution of modes of cell death among different samples compared to their respective control cells in (B) HepG2, (C) A549, and (D) Caco-2.

On the macro scale, the most sensitive samples on the respective cell lines are illustrated in Figure 9. The photos showed the prevailed apoptotic mode of cell death with less necrotic changes.

#### Dual topoisomerase I/II inhibitory assay

Since our main target for the recent study is to discover potent dual Topo I/II inhibitors, the highly active cytotoxic members were subjected to further assessment to measure their Topo I and Topo II inhibitory effects. Camptothecin, the potent Topo I inhibitor, and Dox, the potent Topo II inhibitor, were used as references in this study. Thus, compounds **5b** and **7d** in addition to their nano complexes **5b NPs** and **7d NPs** were evaluated for their enzyme inhibitory effects.

**Figure 10.**
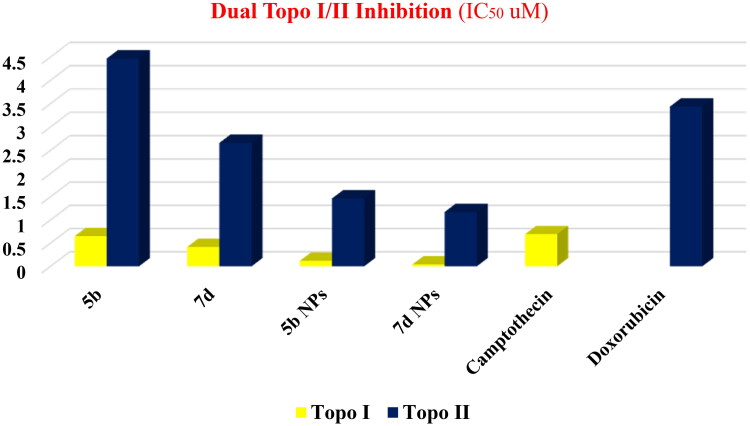
The inhibitory effects of the assessed compounds (**5b**, **7d**, **5b NPs**, and **7d NPs**) against Topo I and Topo II compared to the reference drugs.

With regard to the Topo I enzyme, members **5b** and **7d** exhibited excellent inhibitory activities with similar and better IC_50_ values (0.651 and 0.419 µM), respectively, compared to camptothecin (0.694 µM). However, a drastic increase in the inhibitory activity was noticed regarding the nano complexes **5b NPs** and **7d NPs**. Compounds **5b NPs** and **7d NPs** were 5.8-fold and 16.5-fold more active than camptothecin with IC_50_ values of 0.119 and 0.042 µM, respectively (Figure 10).

Concerning the Topo II enzyme, results also confirmed the potentiality of the tested members as congener **7d** was more active than Dox with IC_50_ of 2.66 µM while its nano complex **7d NPs** showed a potential inhibitory effect with IC_50_ value of 1.172 µM. In contrast, compound **5b** was slightly less potent than Dox with an IC_50_ of 4.469 µM. Nevertheless, member **5b NPs** possessed a higher inhibitory effect than that of Dox with an IC_50_ value of 1.464 µM (Figure 10).

#### DNA intercalation assay

**Figure 11.**
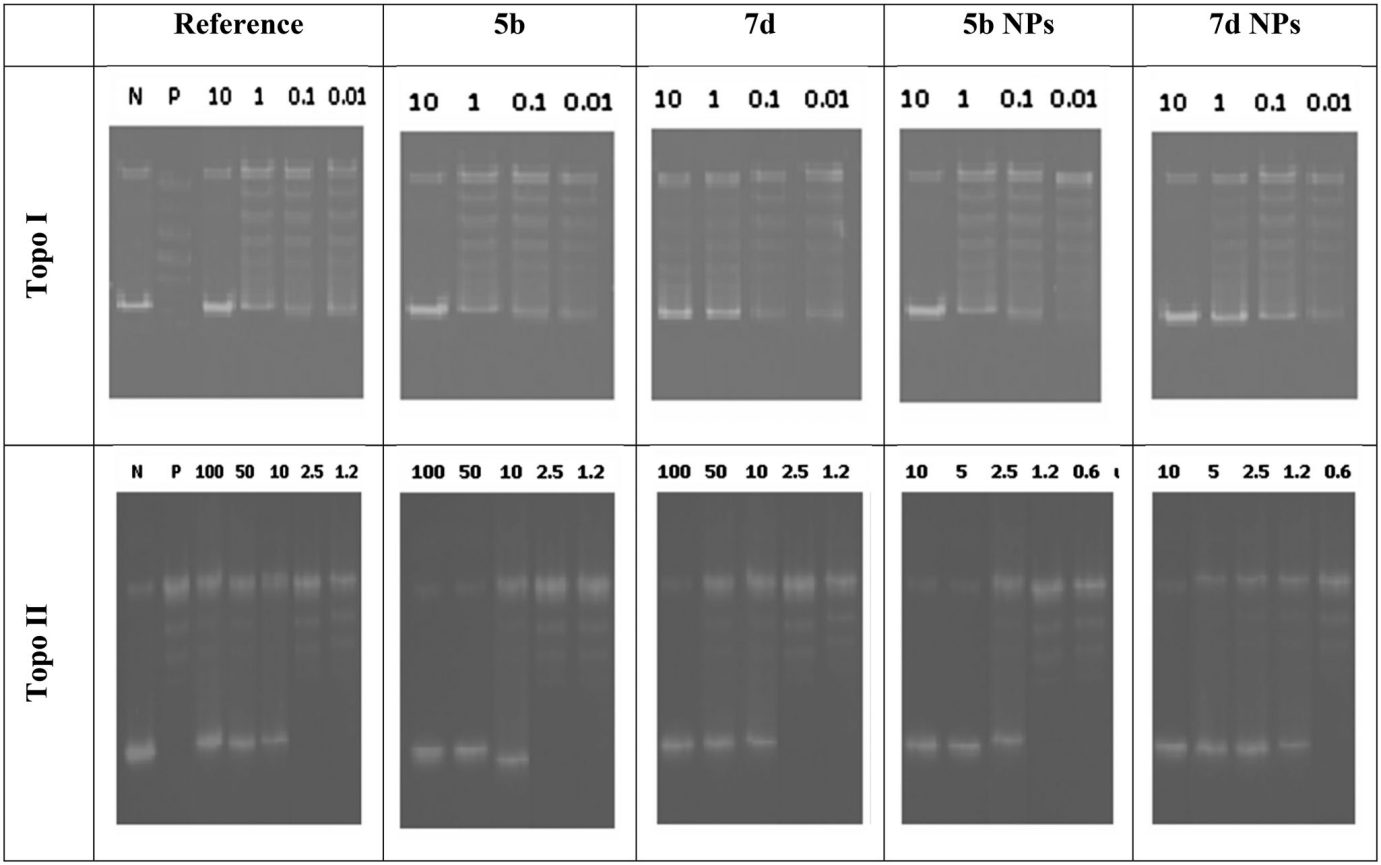
DNA-fragmentation gel images of the most active compounds (**5b** and **7d**) and their nanoforms (**5b NPs** and **7d NPs**) at various concentrations.

The ability of the designed members to intercalate onto the DNA molecules and increase the levels of Topo I–DNA as well as Topo II-DNA cleavage complexes by stabilising the enzymes–DNA covalent complexes were also assayed. Thus, the amount of linear DNA formed by compounds **5b** and **7d**, in addition to their nanoforms **5b NPs** and **7d NPs**, was measured. Even at low concentrations, the tested members caused considerable formation of linear DNA. As so, the designed congeners were then considered promising DNA intercalating members (Figure 11).

#### Cell cycle analysis

The potent cytotoxic activity of congener **7d NPs**, besides its superior dual Topo I/Topo II inhibitory effects, motivated us to perform a cell cycle analysis to explore the specific phase at which **7d NPs** can halt the cell cycle. With this respect, the most sensitive A549 cells were treated with compound **7d NPs** at a concentration equivalent to that produced the cytotoxic effect (1.9 ± 0.1 µg/mL).

**Figure 12.**
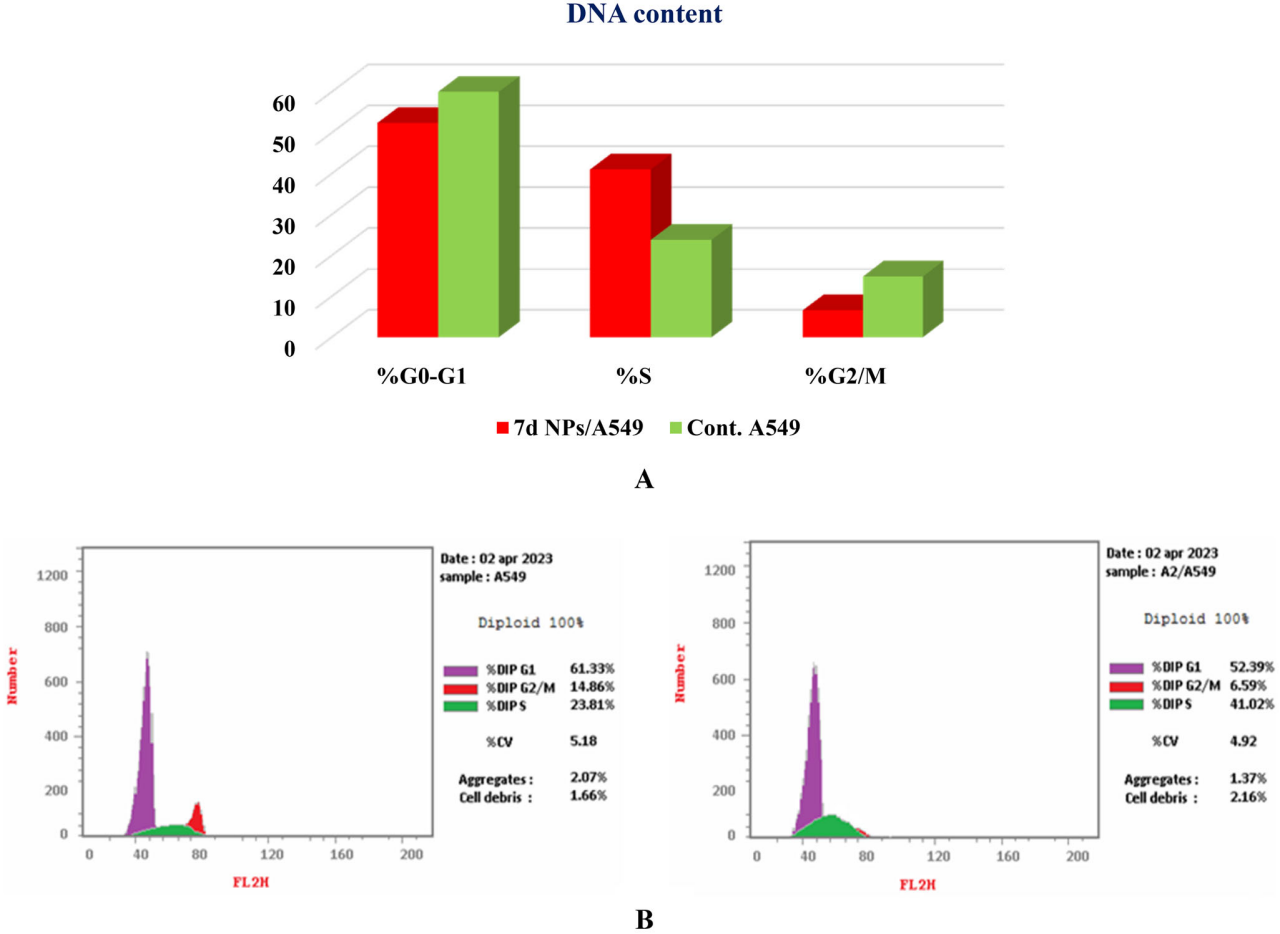
(A) Graphical representation for cell cycle analysis of **7d NPs** treated A549; (B) the histograms show the distribution of cells treated with control (left side) and **7d NPs** (right side).

The obtained results were compatible with the cytotoxicity study. Compound **7d NPs** arrested the cell cycle progression at the S phase as it caused a dramatic increase to 41.02% vs. 23.81%, compared to the untreated cells. Moreover, the percentages of cells at G0–G1 and G2/M phases were relatively reduced from 61.33% and 14.86% in the control cells to 52.39% and 6.59% after incubation with compound **7d NPs**, respectively (Figure 12).

#### Cell apoptosis analysis

**Figure 13.**
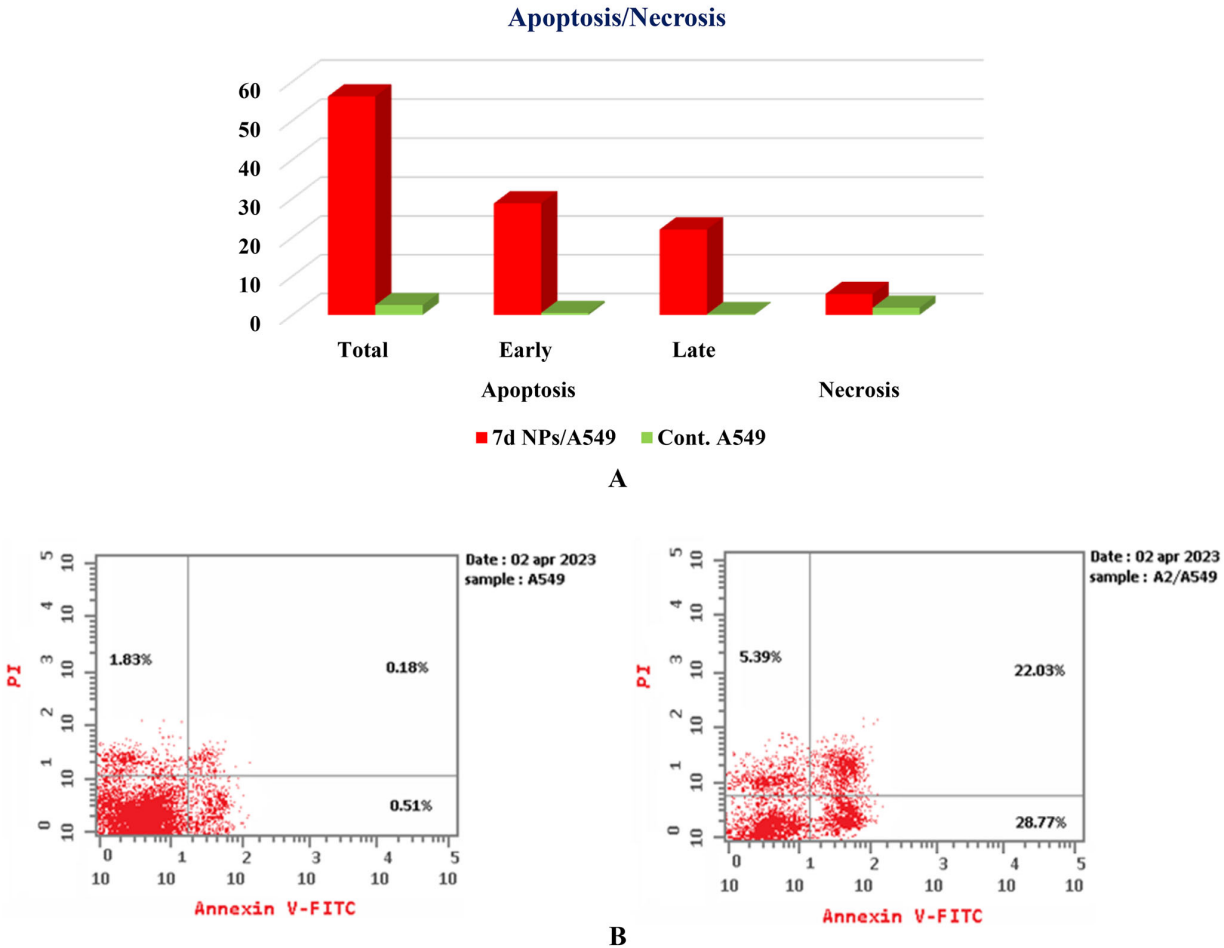
(A) Graphical representation for the % of apoptotic and necrotic cells among control (A549) and **7d NPs** treated cells. (B) The representative histograms show the apoptosis induction of cells treated with control (left side) and **7d NPs** (right side).

The ability of congener **7d NPs** to prompt apoptosis in A549 cells was examined through the Annexin-V/PI staining assay^75^. The present strategy depends upon the incubation of A549 cells with the tested member, **7d NPs**, at the compound’s IC_50_ concentration (1.9 ± 0.1 µg/mL) for 24 h. Based on the given results, **7d NPs** caused a marked induction of apoptosis in the treated cells by a percentage exceeding 56%. In particular, a significant increase in both early and late apoptotic populations from 0.51% and 0.18% in the control cells to 28.77% and 22.03% in the **7d NPs** treated cells confirmed that its cytotoxic activity is associated with a potent apoptotic effect (Figure 13).

### Structure–activity relationship (SAR)

**Figure 14.**
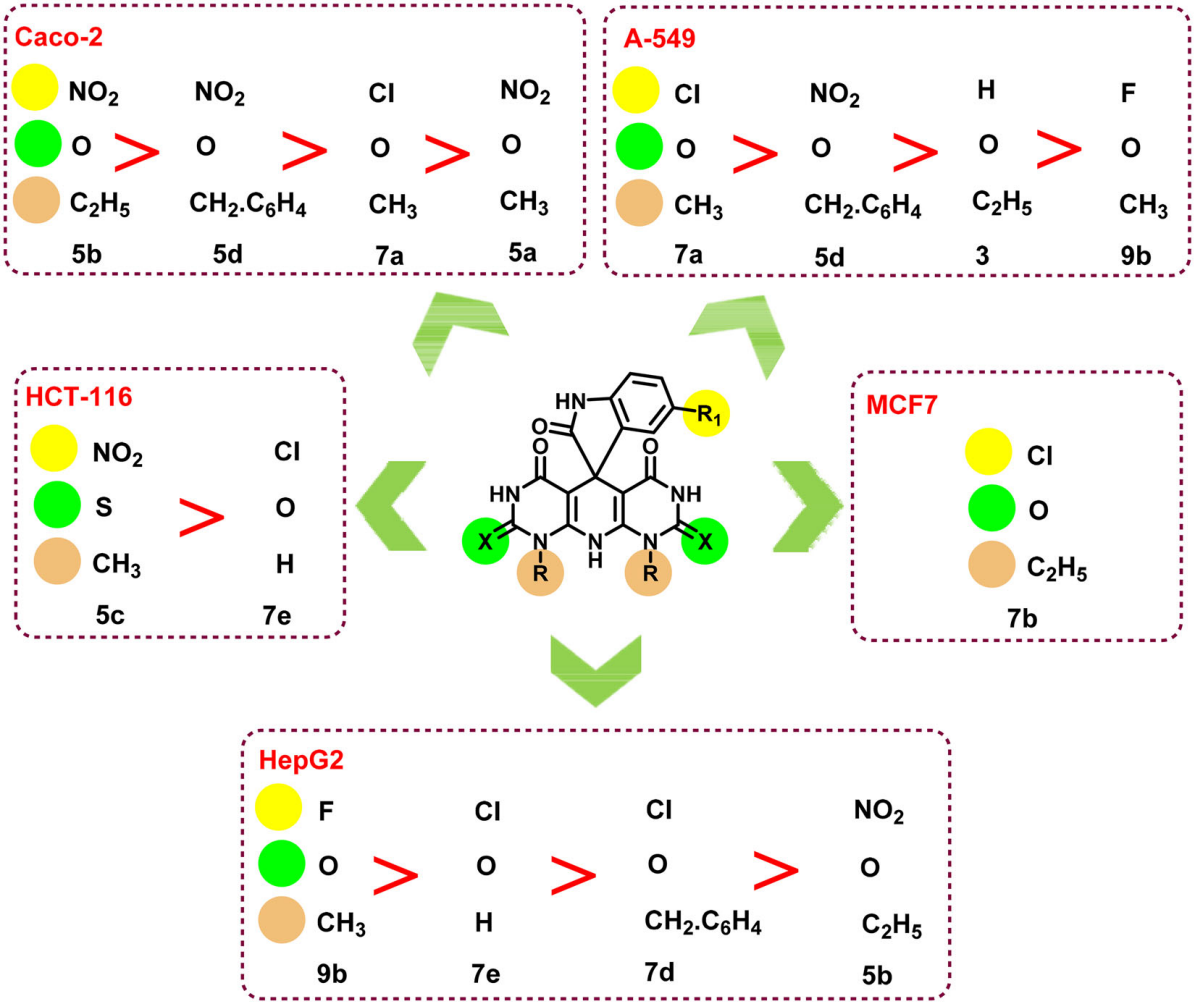
SAR of the tested compounds (**3**, **5a-e**, **7a**–**e**, and **9a,b**).

Observation of the obtained biological findings clarified different notes about the SAR of the designed candidates (Figure 14):

- HepG2, Caco-2, and A-549 cell lines were the most sensitive cells to the examined members among the tested cell lines. As nine members out of 13 were active against HepG2 and Caco-2 cells while seven members were potent against A-549 cells.
- Two compounds were active regarding HCT-116 cells and only one congener of the tested members was active against MCF7 cells.
- The SeNPs complexes **5b NPs** and **7d NPs** exhibited a dramatic increase in the cytotoxic activity against almost all the examined cell lines.
- The fluorinated member **9b** possessed a superior cytotoxic activity regarding HepG2 cells followed by the chlorinated members **7d** and **7e**, which were more potent than the nitro compound **5b**.
- In contrast, the nitro derivatives **5b** and **5d** showed better cytotoxic activities than that of the chlorinated member **7a** against Caco-2 cells.
- The same observation was also reported for HCT-116 cells, as the nitro derivative **5c** was more cytotoxic than the chlorinated member **7e**.
- Concerning A-549 cells, the descending order of activity of the tested members was **7a** > **5d** > **3** > **9b**.
- However, comparing the Topo I/Topo II inhibitory activities as well as the DNA intercalating potential of compounds **5b** and **7d** to the **5b NPs** and **7d NPs** revealed a tremendous increase in the activity of the nano complexes over the native members.

### Molecular docking studies

Two molecular docking studies of the novel designed members (**3**, **5a**–**e**, **7a**–**e**, and **9a,b**) were performed to investigate their potential activities against the human DNA in complex with both Topo I (https://www.rcsb.org/structure/1T8I) and Topo II (https://www.rcsb.org/structure/3QX3) targets, respectively. First, the receptor pockets of the aforementioned targets were studied to clarify the binding mode and accordingly the most important nucleotides and amino acids within each. The co-crystallised poison of the human DNA in complex with Topo I enzyme (camptothecin) was able to bind Asp533, Arg488, DT10, DA113, DC112, and TGP11. Besides, the co-crystallised poison of the human DNA in complex with Topo II enzyme (etoposide) interacted with Gln778, Met782, Asp479, DA12, and DG13. Moreover, each docked co-crystallised ligand was inserted as a reference and showed a similar binding mode as the native one indicating a valid molecular docking software^76^. Herein, the superior anticancer members (**5b** and **7d**) were selected for further investigation.

Both **5b** and **7d** candidates recorded very promising binding scores towards the human DNA in complex with both Topo I and Topo II targets, which were comparable to the co-crystallised ligand in each process. They showed interaction scores of (–9.08 and −7.50 kcal/mol) and (–9.73 and −8.99 kcal/mol), respectively, with respect to camptothecin (–10.24 kcal/mol) and etoposide (–10.84 kcal/mol). All root mean square deviation values were in the range of (1.09–1.56 Å) indicating valid selected poses^77^.

Compound **5b** within the Topo I binding site was able to interact with DA113 with a hydrogen bond and DC112 with a pi–pi interaction. However, compound **7d** described three interactions with DA113 (hydrogen bond), DA113 (pi–pi bond), and TGP11 (pi–pi bond) as depicted in Table 4. Moreover, compound **5b** within the Topo II binding site was found to bind both DA12 (hydrogen bond) and DG13 (hydrogen-pi bond). Furthermore, compound **7d** bound DG13 and His775 with two hydrogen bonds as represented in Table 4.

**Table 4.** 2D interactions, 3D interactions, and 3D positioning of **5b** and **7d** within the binding pockets of the human DNA in complex with Topo I (1T8I) and Topo II (3QX3) target receptors.

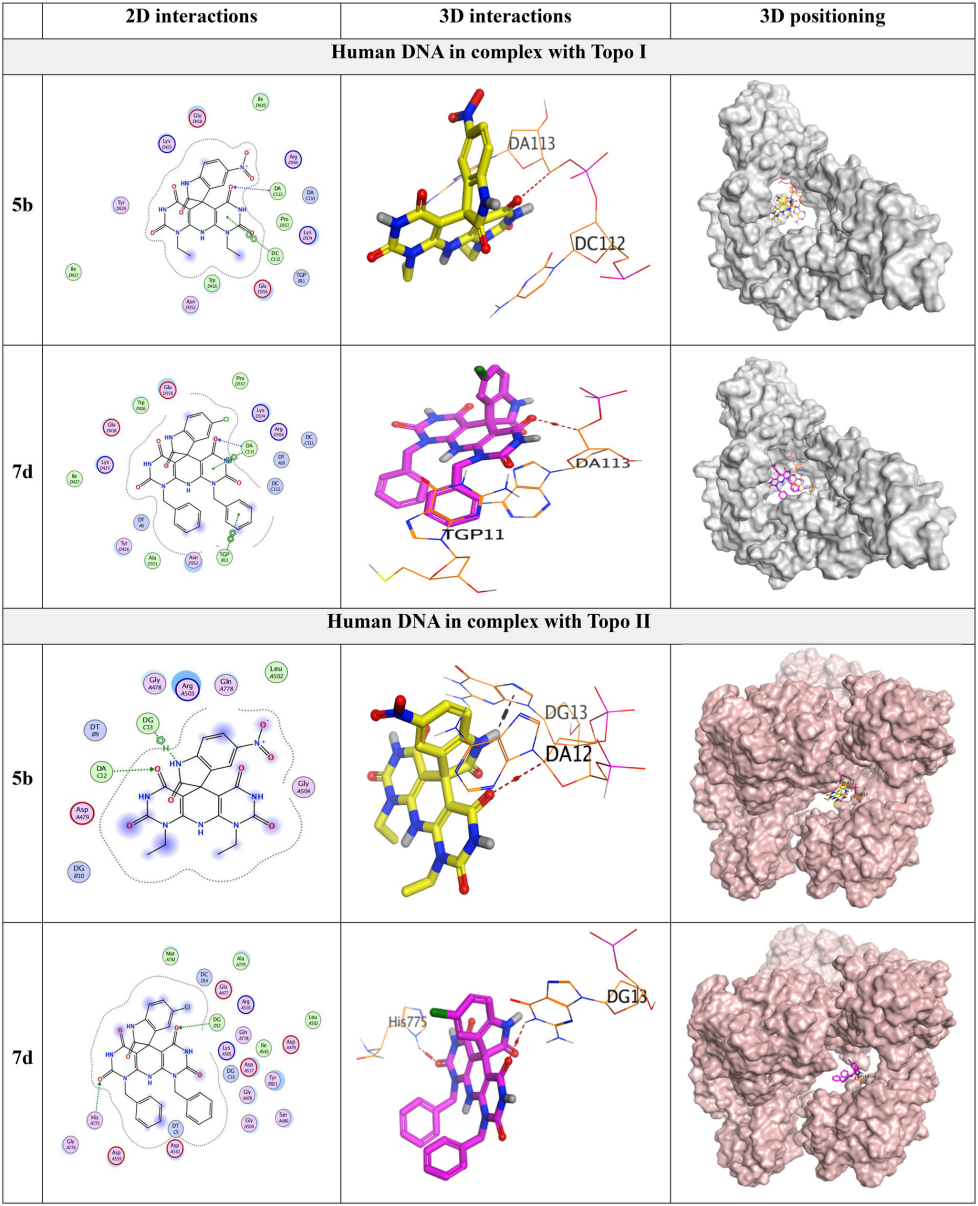			

According to the above results, we can observe the very promising scores and binding modes of both compounds (especially **7d**) to be recommended as a potential DNA intercalator with dual Topo I/Topo II inhibitory activities.

It is worth mentioning that the C═S functional group of both **5c** and **7c** compounds contributed largely to the interactions with the key amino acids of Topo I and Topo II receptors indicating their great importance (Supporting information).

## Conclusions

To find potent multitarget Topo I/II inhibitors, a new wave of compounds was constructed and synthesised based on the hybridisation of spirooxindoles and pyrimidines. The designed compounds were also constructed to act as DNA intercalators. Thirteen congeners were synthesised and *in vitro* assessed for their cytotoxic effects against five human cancer cell lines, HepG2, HCT-116, Caco-2, A-549, and MCF7. Considerable cytotoxic effects were observed regarding the tested cells. However, better cytotoxic compounds were selected for the synthesis of *in situ* SeNPs to enhance their activity. The nanoform compounds **5b NPs** and **7d NPs** exhibited excellent antitumor activities towards the tested cells (IC_50_ values ranging from 1.9 ± 0.1 and 18.9 ± 2.4 µg/mL). Meanwhile, the former two nanoform members were examined for their Topo I and Topo II inhibition effects. Compounds **5b NPs** and **7d NPs** exhibited superior activities against Topo I (IC_50_ = 0.119 and 0.042 µM, respectively). Also, they inhibited Topo II (IC_50_ values = 4.469 and 1.172 µM, respectively). Furthermore, compound **7d NPs** halted the cell cycle in the S phase. Candidate **7d NPs**, moreover, induced apoptosis in A549 cells. Molecular docking also showed very promising binding scores and binding modes (especially for **7d**) to be recommended as a potential DNA intercalator with dual Topo I/Topo II inhibitory potentials.
